# NHD2-15, a novel antagonist of Growth Factor Receptor-Bound Protein-2 (GRB2), inhibits leukemic proliferation

**DOI:** 10.1371/journal.pone.0236839

**Published:** 2020-08-11

**Authors:** Tina R. Lewis, Jesse Smith, Kallie Griffin, Stephanie Aguiar, Kristen F. Rueb, Natalie Holmberg-Douglas, Ellen M. Sampson, Skylar Tomasetti, Sofia Rodriguez, David L. Stachura, Carolynn C. Arpin

**Affiliations:** 1 Department of Biological Sciences, California State University, Chico, Chico, CA, United States of America; 2 Department of Chemistry and Biochemistry, California State University, Chico, Chico, CA, United States of America; Hungarian Academy of Sciences, HUNGARY

## Abstract

The majority of chronic myeloid leukemia (CML) cases are caused by a chromosomal translocation linking the breakpoint cluster region (BCR) gene to the Abelson murine leukemia viral oncogene-1 (ABL1), creating the mutant fusion protein BCR-ABL1. Downstream of BCR-ABL1 is growth factor receptor-bound protein-2 (GRB2), an intracellular adapter protein that binds to BCR-ABL1 via its src-homology-2 (SH2) domain. This binding constitutively activates growth pathways, downregulates apoptosis, and leads to an over proliferation of immature and dysfunctional myeloid cells. Utilizing novel synthetic methods, we developed four furo-quinoxaline compounds as GRB2 SH2 domain antagonists with the goal of disrupting this leukemogenic signaling. One of the four antagonists, NHD2-15, showed a significant reduction in proliferation of K562 cells, a human BCR-ABL1^+^ leukemic cell line. To elucidate the mode of action of these compounds, various biophysical, *in vitro*, and *in vivo* assays were performed. Surface plasmon resonance (SPR) assays indicated that NHD2-15 antagonized GRB2, binding with a *K*_D_ value of 119 ± 2 μM. Cellulose nitrate (CN) assays indicated that the compound selectively bound the SH2 domain of GRB2. Western blot assays suggested the antagonist downregulated proteins involved in leukemic transformation. Finally, NHD2-15 was nontoxic to primary cells and adult zebrafish, indicating that it may be an effective clinical treatment for CML.

## Introduction

Myeloid cells are leukocytes whose major role is to destroy pathogens invading the body. All mature blood cells, including myeloid cells, originate from a multipotent hematopoietic stem cell (HSC) that must generate blood cells for an organism's lifespan [1–3]. HSCs that generate myeloid cells first differentiate into a common myeloid progenitor (CMP) [4, 5], which is capable of generating other hematopoietic progenitor cell (HPC) types called megakaryocyte-erythroid progenitors (MEPs) and granulocyte-monocyte progenitors (GMPs) [4, 5]. MEPs generate erythrocytes and megakaryocytes, while GMPs further differentiate into the full repertoire of the myeloid lineage. Once myeloid cells are differentiated, they migrate out of the bone marrow and travel through the bloodstream to replace dying cells throughout the body. While the exact lifespan of each myeloid cell type is not known, neutrophils make up 50–70% of all of the circulating leukocytes in humans and have a lifespan of about 5 days [6, 7]. This means that the 10^11^ to 10^12^ neutrophils in an adult human need to be replaced on a constant basis [6]. The regulation of this process is intricate and still not completely understood and when perturbations in the production and differentiation of myeloid cells arise there can be severe clinical complications. Underproduction of myeloid cells can lead to neutropenia, allowing pathogens to invade and damage the host. On the other hand, failure to properly differentiate HPCs can lead to leukemia, where the body generates immature, nonfunctional myeloid precursors that deplete the body’s resources and overwhelm the hematopoietic system.

Chronic myeloid leukemia (CML) is a malignant blood disease affecting the normal growth and development of myeloid cells. The vast majority of CML cases are caused by a chromosomal translocation that links the breakpoint cluster region (BCR) gene on chromosome 22 to the Abelson murine leukemia viral oncogene-1 (ABL1) gene on chromosome 9 [8–12]. Normally, the ABL1 protein is a ubiquitously expressed non-receptor tyrosine kinase that when activated triggers cell proliferation, differentiation, or apoptosis [13]. The function of BCR is less clear but two roles are known. First, BCR acts as a GTPase-activating protein (GAP) by activating Ras-related C3 botulinum toxin substrate 1 (RAC1), a GTPase in the RAS superfamily involved in cell proliferation regulation [14]. BCR also acts as a kinase that activates a wide range of signaling pathways [15]. These pathways increase proliferation and inhibit apoptosis and autophagy [16, 17]. In essence, the inappropriate fusion of BCR to ABL1 creates a constitutively active oncogene that activates numerous growth pathways, downregulates apoptosis, and causes leukemic transformation.

Growth factor receptor-bound protein-2 (GRB2) is an intracellular adapter protein responsible for linking receptor tyrosine kinases (RTKs) to downstream signaling proteins involved in cellular growth and differentiation [18]. Generally, once an extracellular ligand binds to an RTK, it will dimerize and autophosphorylate [19]. Once phosphorylated, the RTK dimer will bind to an adapter protein such as GRB2 [20] which can then bind and activate catalytic proteins. As an adapter protein, GRB2 has multiple binding domains. It is one of many proteins to contain both a src-homology-2 (SH2) binding domain as well as a src-homology-3 (SH3) binding domain [21]. The SH2 binding domain is highly conserved, composed of an approximately 100 amino acid sequence that binds phosphorylated tyrosines. The SH3 binding domain binds proline-rich sequences and is found on proteins with and without catalytic capabilities. It is approximately 50–75 amino acids in length and is also highly conserved [22]. GRB2 is a small protein consisting of one SH2 binding domain and two SH3 binding domains [23]; GRB2’s SH2 domain binds to BCR-ABL1 via a phosphorylated tyrosine residue (Y177) on BCR in the same manner that it binds an activated, dimerized RTK [24]. This BCR-ABL1 binding to GRB2 initiates leukemic transformation [25]. After binding to BCR-ABL1, GRB2’s SH3 domain can bind to GRB2-associated-binding protein 2 (GAB2), as well as Son of Sevenless (SOS) and many others [26]. This secondary binding causes the newly formed BCR-ABL1/GRB2 complex to associate with membrane receptors, binding to their intracellular domains to activate cellular growth, survival, and differentiation pathways while inhibiting apoptosis and autophagy [27].

While targeted small molecule therapies exist for CML it is still deadly, affecting mainly patients over 64 years of age. Imatinib, the first drug developed to fight CML, is a tyrosine kinase inhibitor (TKI) specific to the BCR-ABL1 protein [28–30]. While many other second- and third-generation TKIs now exist to treat CML, many patients still develop resistance to these treatments and succumb to the disease [31, 32]. To block downstream signaling of BCR-ABL1 in a different way, we used a novel synthetic reaction to generate small molecules to block BCR-ABL1’s binding to the GRB2 SH2 domain to inactivate leukemic signaling. To do this, we generated furo-quinoxaline based compounds, which have not been well studied mostly due to a lack of efficient synthetic methods. However, we were able to generate four molecules in sufficient yield to test their effect on preventing leukemic transformation. Out of the four antagonists we created and examined, one compound, NHD2-15 bound the GRB2 SH2 domains with a micromolar *K*_D_ value and inhibited the proliferation and metabolism of human leukemia cells. Importantly, NHD2-15 was non-toxic when administered to adult zebrafish at low concentrations, indicating that it may be a specific and effective treatment for this disease.

## Materials and methods

### General synthetic methods

All air- and moisture-sensitive reactions were conducted under a dry nitrogen atmosphere in oven-dried glassware, using standard syringe/cannula transfer techniques unless otherwise reported. Anhydrous dioxane (Sigma Aldrich) was used directly from a Sure/Seal bottle. Unless otherwise stated, all commercial reagents were purchased from Sigma Aldrich and used without further purification. ^1^H NMR spectra were recorded on a Bruker 400 MHz spectrometer in either CDCl_3_ or DMSO-*d*_6_. Chemical shifts (δ) are reported in parts per million (ppm) after calibration to the internal reference solvent peak (CDCl_3_: 7.26 ppm and DMSO-*d*_6_: 2.50 ppm). Coupling constants (*J*) are reported in Hz. Exact mass was calculated for M+Na using electrospray ionization unless reported otherwise. Using a Hewlett Packard 8453 UV-Vis spectrophotometer, solubility was obtained either by measuring the increase in light scatter upon increasing precipitation via absorbance at 700 nm, or by using absorbance calibration curves at the noted wavelength and then extrapolating concentration from saturated sample absorbance [33].

### Synthesis of 2,3-dihydroxyquinoxaline (1)

Diethyl oxalate (37.7 mL, 277 mmol) was added to neat *o*-phenylenediamine (10.0 g, 92 mmol) and allowed to reflux overnight. After cooling to room temperature, the product was isolated via vacuum filtration and rinsed with ethanol to yield **1** as a grey powder (14.10 g, 94%) that was used without further purification. Spectra matched those previously reported [34].

### Synthesis of 2,3-dichloroquinoxaline (2)

DMF (2.40 mL, 31 mmol) was added to a solution of **1** (10.0 g, 62 mmol) in phosphorus oxychloride (3.35 mL, 370 mmol) and allowed to come to reflux. After refluxing for 1.5 h, the reaction was cooled to room temperature and quenched by the slow addition of ice water. Crude product was isolated via vacuum filtration and rinsed with water. Recrystallization from hexanes gave **2** as white crystals (9.64 g, 79%). Spectra matched those previously reported [35].

### General procedure for furo-quinoxaline synthesis

The *β*-diketone (1.0 equiv) was added to a round bottom flask, followed directly by 60% sodium hydride in mineral oil (2.0 equiv), upon which some fuming occurred. Dioxane (0.02 M solution) was then added, and the mixture was stirred at room temperature for about 5 minutes, during which the solution bubbled. To this enolate solution, **2** (2.0 equiv) was then added, followed by FeSO_4_ (0.1 equiv) that had been previously dried by leaving in a 150°C oven and open to air overnight. The reaction was then left open to air, heated to reflux, and subjected to a 150 V incandescent lamp. When TLC showed full consumption of starting material (usually about 6 h), the dark maroon solution was cooled to room temperature and then quenched by the addition of saturated NH_4_Cl. The solution was transferred to a separatory funnel, rinsed with EtOAc, and washed with NH_4_Cl (3×), then back-extracted aqueous with EtOAc (3×). The combined organics were then washed with brine, dried over Na_2_SO_4_, and concentrated *en vacuo*. Flash column purification over silica yielded the desired solid product.

### Synthesis of NHD2-15

Acetylacetone reacted with sodium hydride, 2, and dried FeSO_4_ in dioxane on a 0.26 mmol scale following the general procedure. Flash column purification over silica with a gradient of 15:1 Hex:EtOAc yielded NHD2-15 as a yellow solid (0.015 g, 26%). ^1^H NMR (400 MHz, CDCl_3_) δ 8.32–8.25 (m, 1H), 8.19–8.12 (m, 1H), 7.84–7.79 (m, 2H), 3.02 (s, 3H), 3.00 (s, 3H). ^13^C NMR (100 MHz, CDCl_3_): δ 172.06, 167.75, 152.78, 142.07, 141.24, 138.39, 132.48, 131.15, 130.87, 129.29, 128.81, 128.76, 128.70, 115.89, 31.48, 15.97. HRMS (ESI) calcd: 249.0640 (for C_13_H_10_N_2_O_2_ + Na), found: 249.0638. Solubility was obtained by use of an absorbance calibration curve at 367.5 nm: 515 ± 6 μM in water and 1.0% DMSO.

### Synthesis of NHD2-92

Methyl acetoacetate reacted with sodium hydride, 2, and dried FeSO_4_ in dioxane on a 0.17 mmol scale following the general procedure. Flash column purification over silica with a gradient of 4:1 Hex:EtOAc yielded NHD2-92 as a yellow solid (0.042 g, 24%). ^1^H NMR (400 MHz, CDCl_3_) δ 8.32–8.24 (m, 1H), 8.10–8.01 (m, 1H), 7.77–7.67 (m, 2H), 4.00 (s, 3H), 2.92 (s, 3H). ^13^C NMR (100 MHz, CDCl_3_): δ 172.74, 162.86, 152.78, 142.38, 140.67, 138.49, 129.60, 129.46, 128.72, 128.67, 109.05, 52.32, 15.77. HRMS (ESI) calcd: 265.0589 (for C_13_H_10_N_2_O_3_ + Na), found: 265.0592. Solubility was obtained by measuring light scatter at 700 nm upon increasing precipitation: >1588 μM in water and 2.0% DMSO.

### Synthesis of NHD2-107

Ethyl benzoylacetate reacted with sodium hydride, 2, and dried FeSO_4_ in dioxane on a 0.23 mmol scale following the general procedure. Flash column purification over silica with a gradient of 7:1 Hex:EtOAc yielded NHD2-107 as a pale yellow solid (0.0.027 g, 37%). ^1^H NMR (400 MHz, CDCl_3_) δ 8.41–8.34 (m, 1H), 8.22–8.09 (m, 3H), 7.83 (dt, *J* = 6.5, 3.5 Hz, 2H), 7.67–7.46 (m, 4H), 4.57 (q, *J* = 7.1 Hz, 2H), 1.47 (t, *J* = 7.1 Hz, 3H). ^13^C NMR (100 MHz, CDCl_3_): δ 165.93, 162.37, 152.85, 142.76, 141.87, 138.99, 132.18, 129.71, 129.68, 129.63, 129.37, 128.70, 128.61, 128.56, 128.15, 108.94, 61.71, 14.18. HRMS (ESI) calcd: 341.0902 (for C_19_H_14_N_2_O_3_ + Na), found: 341.0911. Solubility was obtained by use of an absorbance calibration curve at 247 nm: 130 ± 20 μM in water and 2.0% DMSO.

### Synthesis of NHD2-114

Ethyl acetoacetate reacted with sodium hydride, 2, and dried FeSO_4_ in dioxane on a 0.50 mmol scale following the general procedure. Flash column purification over silica with a gradient of 5:1 Hex:EtOAc yielded NHD2-114 as a pale yellow solid (0.0.027 g, 21%). ^1^H NMR (400 MHz, CDCl_3_) δ 8.31–8.24 (m, 1H), 8.08–8.01 (m, 1H), 7.75–7.68 (m, 2H), 4.46 (q, *J* = 7.1 Hz, 2H), 2.91 (s, 3H), 1.43 (t, *J* = 7.1 Hz, 3H). ^13^C NMR (100 MHz, CDCl_3_): δ 172.06, 162.25, 152.78, 142.44, 141.00, 138.45, 129.71, 129.38, 129.38, 128.62, 109.34, 61.27, 15.86, 14.42. HRMS (ESI) calcd: 279.0746 (for C_14_H_12_N_2_O_3_ + Na), found: 279.0747. Solubility was obtained by use of an absorbance calibration curve at 367.5 nm: 1200 ± 100 μM in water and 5.7% DMSO.

### Surface Plasmon Resonance (SPR) spectroscopy

SPR experiments were performed using a BIAcore™ 3000 instrument (GE Healthcare). A CM5 chip surface was activated with 1:1 *N*-hydroxysulfosuccinimide(NHS)/1-(3-dimethylamino-propyl)-3-ethylcarbodiimide hydrochloride (EDC) immediately followed by ethanolamine blocking. GRB2 was immobilized on a CM5 sensor chip following the manufacturer’s instructions until an immobilization level of ~12 kRU was obtained at a flow rate of 30 μL/min. Each antagonist was then diluted with HBS-EP buffer (0.01 M HEPES pH 7.4, 0.15 M NaCl, 3 mM EDTA, 0.005% v/v Surfactant P20) to final concentrations of 125, 62.5, 31.25, 15.63, 7.81, 3.91, and 0 μM in 0.5% DMSO. Antagonist was then injected into the GRB2-immobilized CM5 sensor chips and the response read. Data analysis was performed using Scrubber 2.0 software. Full sensorgrams and dose-response curves are provided in the S4–S11 Figs.

### Enzyme Linked Immunosorbent Assay (ELISA)

NHD2-15 was resuspended in DMSO at 25 mM. 96-well plates coated with streptavidin (Thermo Fisher) were incubated for 1 h at 37°C with 100 μL of a 100 nM solution of the GRB2 SH2 domain-binding peptide (biotin-Aha-PSpYVNVQN) [36] in TBS buffer (100 mM Tris, 50 mM NaCl, pH 7.5). Then, 800 μL per well of SuperBlock™ Blocking Buffer (Thermo Scientific) was added to each well and incubated for 4 h. NHD2-15 at desired concentrations (60, 30, 15, and 0 μM) was diluted in SuperBlock™ and GST-GRB2 (20 nM, 100 μL per well) and were both added to the desired wells and incubated for 1 h at 37°C. The plates were then rinsed four times using SuperBlock™ and 0.05% Tween 20. Next, 100 μL per well of anti-GST antibody with horseradish peroxidase (HRP) (Invitrogen; dilution of 1:4000 in SuperBlock™ with 0.05% Tween 20) was added, and the plate was then incubated for 1 h at 37°C. The plate was then rinsed four times using SuperBlock™ with 0.05% Tween 20 before being incubated in the presence of 200 μL per well of TMB developing solution at room temperature for approximately 30 s. The reaction was stopped by adding 100 μL of 10% (V/V) sulfuric acid per well. The absorbance reading was taken at 450 nm. An equivalent well with no peptide was utilized to determine the background of this reaction, which was subtracted from the absorbance reading.

### Cellulose Nitrate (CN) filtration assay

NHD2-15 was resuspended in DMSO at 25 mM. CN filtration assays were performed with 4 μM GRB2 incubated with either 8 μM SH2 domain inhibitor (K_D_ = 400 nM) [37], 16 μM SH3 inhibitor (K_D_ = 4 μM) [38], both inhibitors, or neither inhibitor (all from Santa Cruz Biotechnology) for 0.5 h in TBS with 0.5% DMSO. Afterwards, antagonist was added at a final concentration of 25 μM and allowed to incubate for an additional 0.5 h at room temperature. The solution was then passed through a 0.2 μm CN syringe filter (Thermo Fisher) and was analyzed via an HP 6890 series gas chromatograph (GC) flame ionization detector (FID) with an omegawax 250 column (Sigma Aldrich). Standard calibration curves were obtained via GC-FID analysis of varying drug concentrations of 100, 75, 50, 25, and 0 μM. Response versus concentration was plotted and linear regression analysis utilized to determine the final concentration of antagonist in the filtrate solution.

### Western blotting

Protein isolated from whole cell lysate was separated on 4–20% acrylamide gel (Mini-Protean GTX, BioRad). Protein was transferred to PVDF membranes and blocked with 10% milk for 1 h. Gels were stained post-transfer using Coomassie stain to visualize loaded protein levels. Blots were probed with anti-GRB2 antibodies (dilution of 1:5,000; Abcam, Ab32037), anti-phosho-CRKL antibodies (dilution of 1:1000; Cell Signaling Technology Inc., 3181S), and anti-phospho-c-ABL1 antibodies (dilution of 1:1000; Cell Signaling Technology Inc., 2868S). Anti-GAPDH antibodies (dilution of 1:15,000; Abcam, Ab181602) were used as a loading control. Goat anti-rabbit IgG coupled to HRP secondary antibodies were used at 1:10,000 dilution (Abcam). Blots were stained with Ponceau stain to confirm loading amounts. ImageJ was used to quantitate pixel density.

### Cell culture

K562 cells were obtained from the American Type Tissue Collection (ATCC, catalog number CCL-243) and maintained in RPMI 1640 media (Corning) with 10% fetal bovine serum (FBS), 1% penicillin/streptomycin, and 1% L-glutamine. Normal primary human peripheral blood mononuclear cells (PBMCs) were obtained from ATCC (catalog number PCS-800-011) and maintained in RPMI 1640 media (Corning) with 10% fetal bovine serum (FBS), 1% penicillin/streptomycin, and 1% L-glutamine. HEK293FT cells were obtained from ATCC (catalog number CRL-3216) and maintained in DMEM (Corning) with 10% FBS, 1% penicillin/streptomycin, and 1% L-glutamine. All mammalian cell lines were maintained at 37°C with 5% CO_2_. Primary Zebrafish Kidney Stromal (ZKS) cells were maintained and cultured as previously described [39]. All cell lines were monitored visually to confirm their morphology and tested for mycoplasma contamination via PCR analysis before use.

### Cell proliferation

K562 cells were plated at 1 x 10^5^ cells/well in a 12-well plate and incubated for 72 h with NHD2-15, DMSO vehicle control, or imatinib. PBMCs were plated at 1 x 10^5^ cells/well in a 12-well plate and incubated for 48 h with NHD2-15 or imatinib. ZKS were plated at 1 x 10^5^ cells/well in a 12-well plate and incubated for 72 h with NHD2-15 or imatinib. Cells were enumerated with a Cell Counter and CytoSMART Cloud Application (Corning Inc., Corning NY).

### CellTiter 96 metabolic assay

K562 cells were resuspended at 5 x 10^5^ cells/mL; 100 μL of cell suspension was placed in individual wells of a 96-well plate with NHD2-15, DMSO vehicle control, or imatinib. Cells were cultured for 48 h at 37°C in 5% CO_2_, 15 μL CellTiter dye was added, the reaction was incubated for 2 h and then 100 μL stop solution was added. Plates were incubated for an additional 24 h at 37°C and absorbance at 570 nm was assessed.

### Zebrafish husbandry and care

Zebrafish were mated, staged, and raised as described [40] and maintained in accordance with California State University (CSU), Chico Institutional Animal Care and Use Committee (IACUC) guidelines. The CSU Chico IACUC approved these studies before they were performed. Personnel were trained in animal care by taking the online Citi Program training course entitled “Working With Zebrafish (*Danio rerio*) in Research Settings” (https://www.citiprogram.org). AB wild type (wt) fish were utilized for this study. Zebrafish were housed in a 700L recirculating zebrafish aquarium system (Aquatic Enterprises, Seattle, WA) regulated by a Profilux 3 Outdoor module that regulated salinity, pH, and temperature (GHL International, Kaiserslautern, Germany) 24-hours-a day. The facility was illuminated on a 14-hour light/ 10-hour dark cycle. Zebrafish were fed once a day with hatched brine shrimp (Brine Shrimp Direct, Ogden, UT) and once a day with Gemma micro 300 (Skretting, Westbrook, ME).

### Zebrafish toxicity

Adult zebrafish (6-months-old), raised at our animal facility at CSU Chico, were individually placed in a 500 mL breeding tank with 15 μM of NHD2-15 (n = 12, 6 males and 6 females), DMSO vehicle control (n = 8, 4 males and 4 females), or 2 μM imatinib (n = 8, 4 males and 4 females) and incubated for 3 days. Another experiment utilized 30 μM of NHD2-15 (n = 8, 4 males and 4 females), and another experiment was performed whereby 30 μM of NHD2-15 (n = 4, 2 males and 2 females) was added to the water for two hours before placing the fish into fresh system water. Every 24 h fish were fed. Every 2 hours fish were analyzed for signs of distress, including erratic swimming, ruffled gills and fins, lack of movement, and labored breathing. If fish showed any signs of distress they would have been immediately euthanized by an overdose of tricane methanesulfonate (MS-222), but that never occurred.

### Statistical methods

Statistical analyses were performed in Microsoft Excel. To discern statistical difference, data were analyzed using an unpaired two-tailed Student’s T test assuming unequal variance. All raw data from these studies are supplied in Supporting Information.

## Results

### Library synthesis

**Fig 1:**
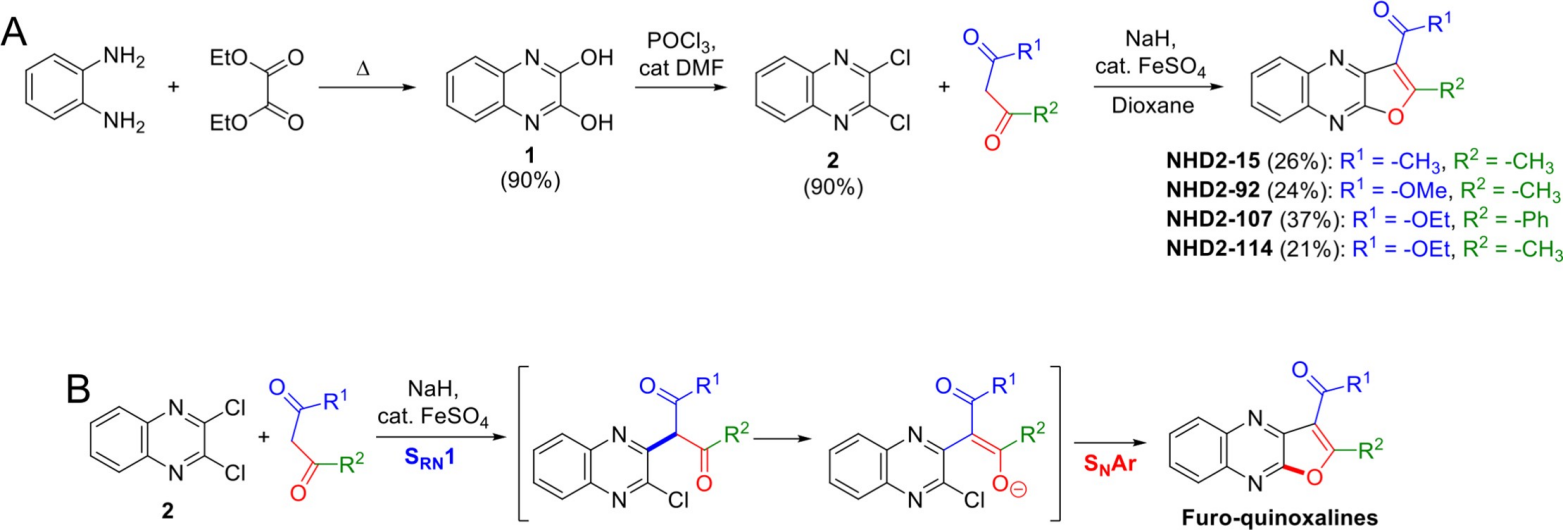
Synthetic preparation of furo-quinoxalines NHD2-15, NHD2-92, NHD2-107, and NHD2-114. (A) Library preparation consisted of only three synthetic steps, the last of which is a novel reaction. (B) Proposed mechanism of the furo-quinoxaline formation: a new domino reaction beginning with an S_RN_1 *C*-arylation followed by *O*-arylation via S_N_Ar.

As shown in Fig 1A, synthetic preparation of the furo-quinoxaline library commenced with the acyl substitution of diethyl malonate with *o*-phenylenediamine to give 2,3-dihydroxyquinoxaline (Fig 1A, structure **1**). Treatment of **1** with phosphorus oxychloride and catalytic DMF yielded the dichloro-substituted quinoxaline (Fig 1A, structure **2**). Finally, deprotonation of the *β*-diketone with sodium hydride followed by addition of **2** and catalytic iron (II) sulfate yielded the desired final compounds. A proposed mechanism for this novel reaction is a tandem S_RN_1 *C*-arylation followed by *O*-arylation via S_N_Ar as described in Fig 1B. These reactions generated four compounds that we refer to as NHD2-15, NHD2-92, NHD2-107, and NHD2-114.

### GRB2 binding as assessed by SPR

To quantitatively assess the binding affinity of our antagonists to GRB2, SPR spectroscopy was used to measure the equilibrium dissociation constant, *K*_D_, of each antagonist for GRB2. All compounds had *K*_D_ values in the micromolar to millimolar range (S1 Table). NHD2-15 had the highest affinity for GRB2 with a *K*_D_ of 119 ± 2 μM. NHD2-114 also had micromolar affinity with a *K*_D_ of 440 ± 7 μM. NHD2-92 and NHD2-107 displayed weaker affinity in the millimolar range (1000 ± 20 μM and 3400 ± 100 μM, respectively). While SPR analysis indicated that all library molecules bound to GRB2, the experimental conditions were sub-par for NHD2-114, NHD2-92, and NHD2-107. Therefore, we performed all subsequent assays with NHD2-15, which had the most potent binding (see all analysis of SPR in S4–S11 Figs).

### GRB2 SH2 domain binding

**Fig 2:**
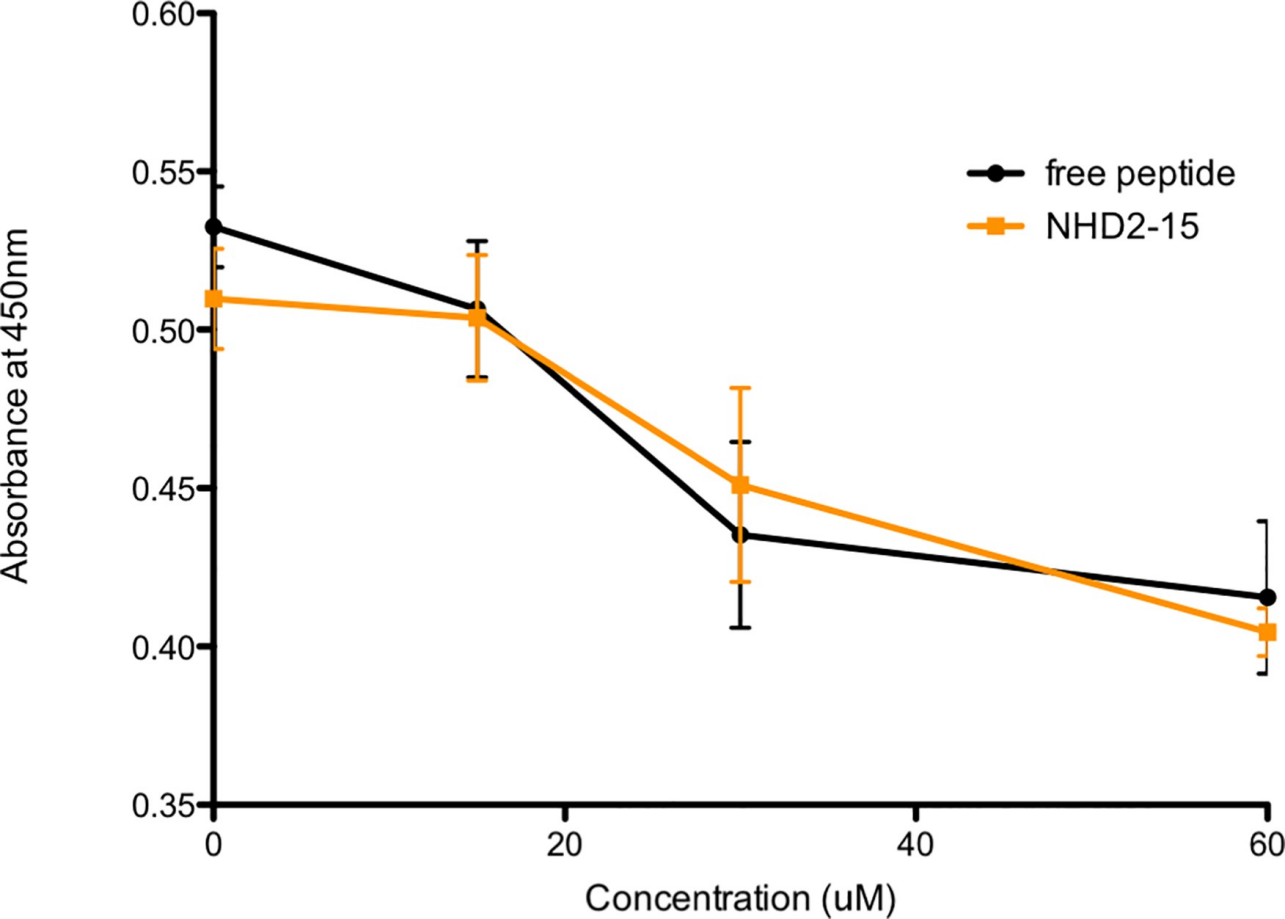
NHD2-15 binds similarly to GRB2’s natural ligand. ELISA results with increasing concentrations of GRB2 antagonist and free peptide were performed. Peptide with a biotin tag was added to streptavidin-coated wells, and GST-tagged GRB2 and NHD2-15 were incubated together, allowing GRB2 to bind either the peptide or the antagonist. Wells were rinsed, and an anti-GST antibody coupled with HRP was introduced. TMB was added and the reaction was stopped with sulfuric acid and analyzed via UV-Vis at 450 nm. Data points are the mean, error bars denote SD. n = 4 for all points.

To confirm that NHD2-15 bound to the SH2 domain of GRB2, ELISA experiments were performed, evaluating how well NHD2-15 competitively displaced a native GRB2 SH2 domain-binding peptide [36]. NHD2-15 bound the GRB2 SH2 domain similarly to increasing amounts of native peptide (Fig 2). These ELISA experiments indicated that NHD2-15 bound to the GRB2 SH2 domain as well as the domain’s native binding peptide at all concentrations examined.

### GRB2 domain binding specificity

**Fig 3:**
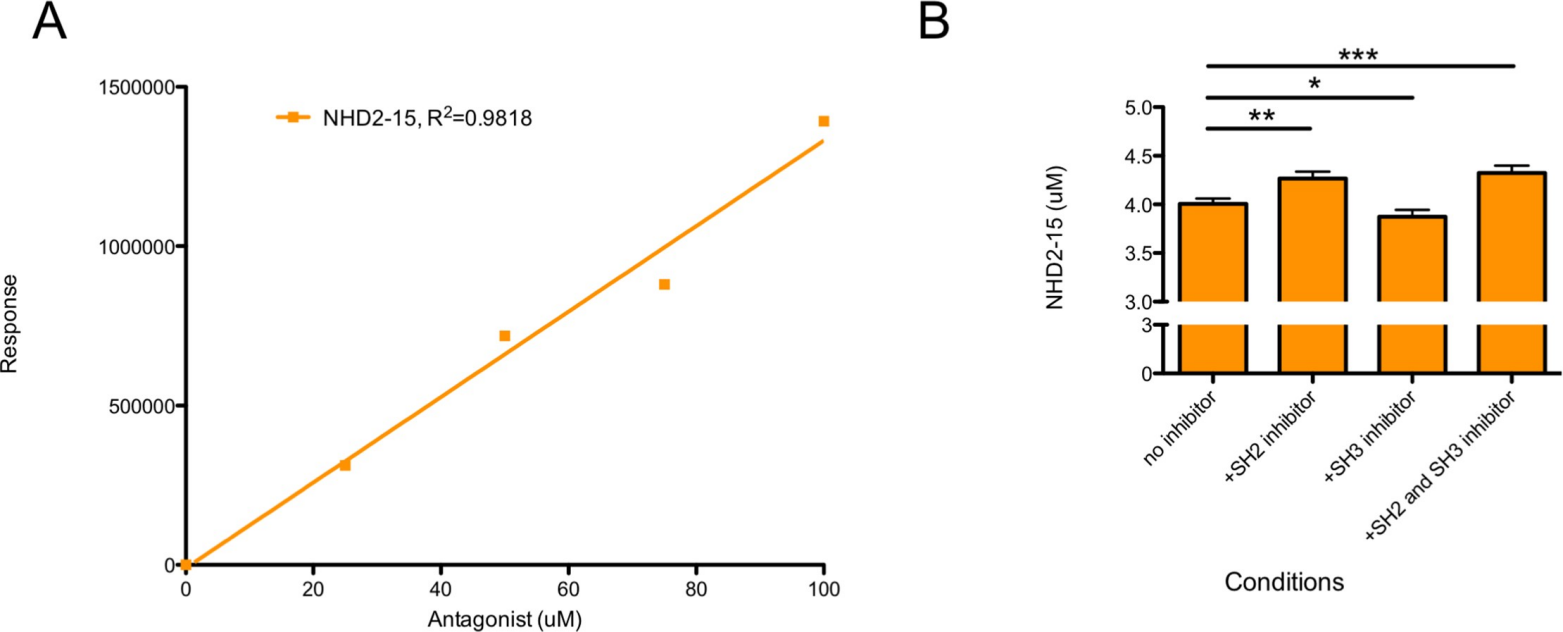
NHD2-15 binds to GRB2’s SH2 domain. (A) NHD2-15 was diluted to 100, 75, 50, and 25 μM and analyzed with GC-FID. The response vs. concentration was plotted and a linear regression line constructed. The equation of the line was used to calculate the concentrations of NHD2-15 in (B). (B) 4 μM of GRB2 was incubated in either 8 μM SH2 domain inhibitor, 16 μM SH3 inhibitor, both inhibitors, or neither. After incubation, NHD2-15 was added and incubated, creating a final concentration of 25 μM before passing through a 0.2 μm cellulose nitrate syringe filter. The filtrate was then analyzed via GC-FID. * p = 0.029, ** p = 0.001; *** p = 0.0007; n = 4; error bars = SD.

To evaluate the specificity of NHD2-15 to bind the GRB2 SH2 domain as opposed to the protein’s SH3 domains, CN filtration experiments were utilized. After incubation with a specific GRB2 domain inhibitor of known potent binding [36], NHD2-15 was incubated with GRB2. The mixture was then passed through a CN filter which binds peptides but allows most small amphipathic small molecules to pass through. The filtrate was then analyzed by GC-FID, compared to a calibration curve (Fig 3A), and the concentration of drug in the filtrate was determined. With the GRB2 SH2 domain occupied, the concentration of NHD2-15 in the filtrate increased when compared to those detected in the absence of the domain inhibitor (Fig 3B). When the protein’s SH3 domains were blocked but its SH2 domain was not, the concentration of NHD2-15 in the filtrate was comparable to those detected without an inhibitor. Combined, these results indicate that NHD2-15 bound the GRB2 SH2 domain with specificity over the protein’s SH3 domains.

### GRB2 is highly expressed in leukemic K562 cells

**Fig 4:**
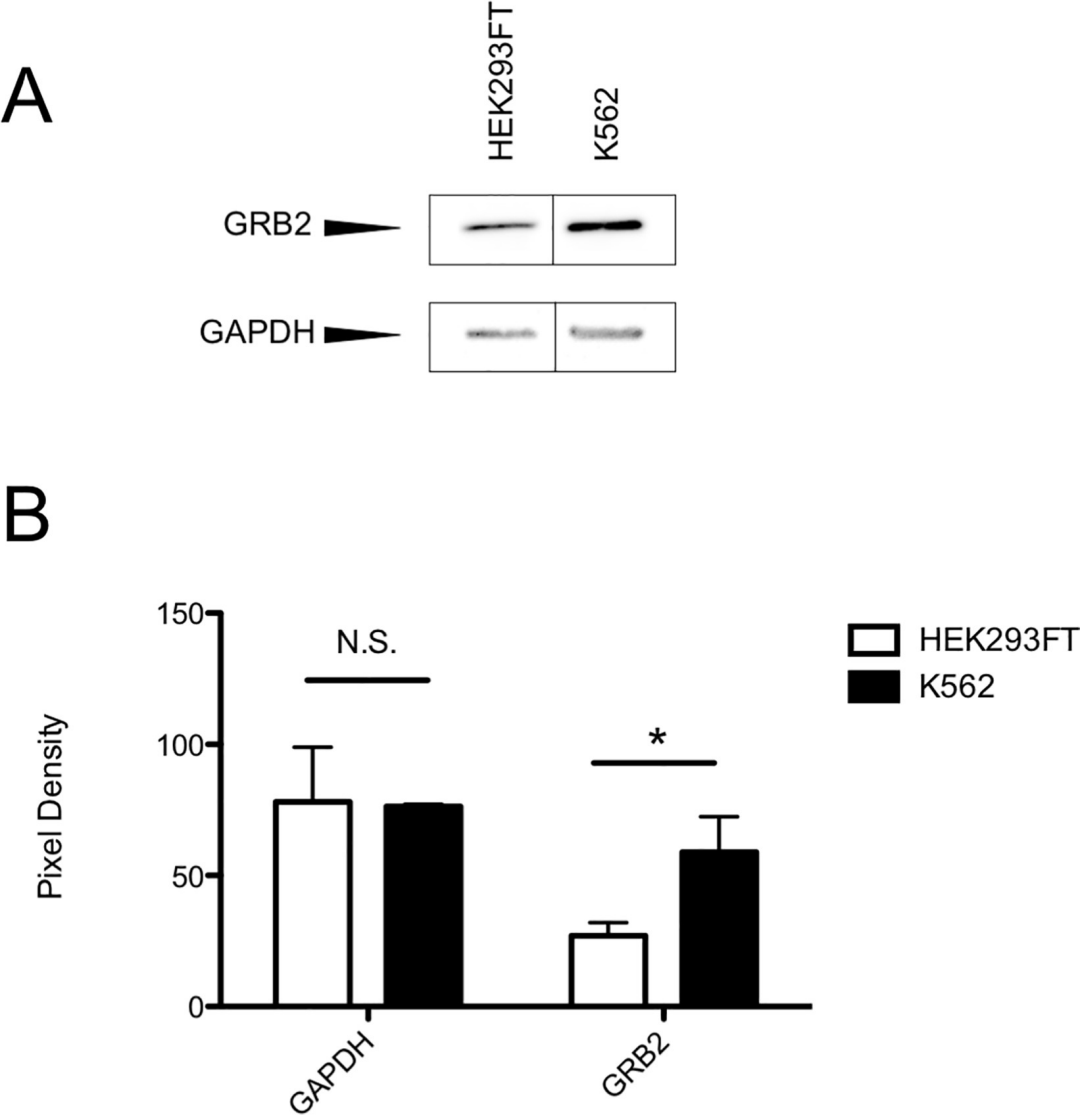
GRB2 is overexpressed in K562 cells. (A) Representative western blot of 10 μg total cell lysate isolated from HEK293FT and K562 cells probed for GRB2 (top) and GAPDH (bottom). (B) Densitometry of HEK293FT (white bars) and K562 (black bars) western blots probed for GAPDH (left) and GRB2 (right). * p = 0.04, n = 3; error bars = SD; N.S., not significant (p = 0.89).

To develop a cellular model to study the effect of NHD2-15 on cell growth, we investigated the human BCR-ABL1^+^ K562 leukemic cell line. To determine if K562 cells had high expression of GRB2, we compared the levels of GRB2 in K562 cells to that of non-malignant cells. Western blotting of total cell lysate from K562 cells and human embryonic kidney cells (HEK293FT) showed elevated expression of GRB2 in K562 cells relative to HEK293FT cells (Fig 4). Data obtained from the Cancer Cell Line Encyclopedia (CCLE, https://portals.broadinstitute.org/ccle) also indicated that GRB2 mRNA expression was elevated in a variety of human cancer cell lines, especially commonly utilized leukemic cell lines like Kasumi-1, HL-60, THP-1, K562, and RS4;11. These results indicated that elevated GRB2 mRNA expression levels are a feature of CML as well as other human cancers, and likely play a role in their proliferative capacity.

### NHD2-15 inhibits growth of K562 cells

**Fig 5:**
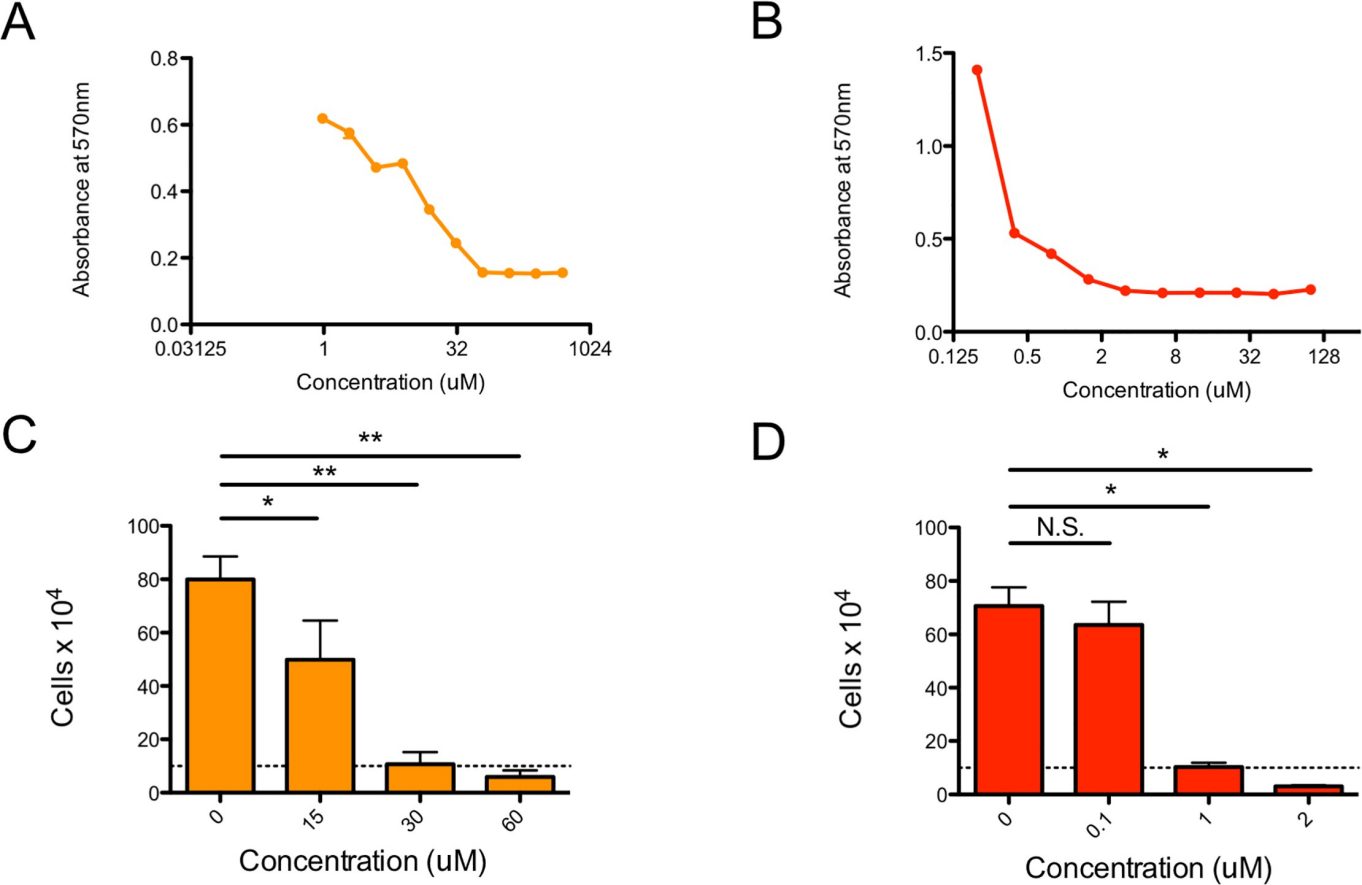
Treatment with NHD2-15 reduces metabolism and proliferation of K562 cells in a dose-dependent manner. (A) 1×10^5^ K652 cells were incubated with increasing amount of NHD2-15. Cells were also cultured with imatinib as a positive control (B). After 72 h, a CellTiter 96 Assay was performed and absorbance at 570 nm was assessed. Filled circles represent the mean, and error bars represent SD; n = 3 for all trials. (C) 1×10^5^ K652 cells were incubated with 0, 15, 30, or 60 μM of NHD2-15. Bars represent the mean, and error bars represent SD; * p < 0.017, ** p < 0.001; n = 4. Cells were also cultured with 0, 0.1, 1, or 2 μM of imatinib as a positive control (D). After 72 hours, cells were enumerated by trypan blue exclusion. Bars represent the mean, and error bars represent SD; N.S. denotes no significance (p = 0.32), * p < 0.003; n = 3. Dashed line denotes starting amount of cells.

To determine if NHD2-15 would inhibit the growth of K562 cells, we first performed a CellTiter 96 assay, which measures the metabolic activity of cells. Exposing K562 cells to increasing amounts of NHD2-15 decreased their metabolic activity (Fig 5A), especially in the 15–120 μM range. Imatinib was run as a positive control, and reduced K562 cell metabolism at 1–2 μM, as previously reported [41] (Fig 5B). Overall, NHD2-15 had an inhibitory effect on the metabolic activity of K562 cells.

To validate our CellTiter 96 results, we also performed a cell proliferation assay. K562 cells were exposed to 15–60 μM of NHD2-15 and enumerated with a cell counter after 72 h of exposure (Fig 5C). As the concentration of antagonists increased, cell growth was reduced (Fig 5C), correlating with the data in Fig 5A. Imatinib effectively killed the K562 cells at a concentration of 1–2 μM (Fig 5D). Overall, NHD2-15 was effective at reducing the growth of K562 cells in culture.

### NHD2-15 does not inhibit growth of PBMCs

To determine if NHD2-15 specifically inhibited leukemic blood cells versus normal blood cells, we exposed untransformed, primary PBMCs to NHD2-15 and imatinib. PBMCs are post-mitotic, and do not proliferate in culture without exogenous cytokine addition and die within a week of culture under normal *in vitro* conditions. PBMCs were exposed to 15–60 μM of NHD2-15 and enumerated with a cell counter after 48 h of exposure (S1 Fig). As the concentration of NHD2-15 increased, cell growth remained constant (S1A Fig), indicating that it did not inhibit the growth of healthy human blood cells. As a control, PBMCs were also exposed to imatinib, which also had no negative effect on cell growth (S1B Fig**)**. Together, these data indicate that NHD2-15 is a selective growth inhibitor of leukemic cells and not untransformed human blood cells.

### NHD2-15 does not inhibit growth of ZKS cells

To further illustrate that NHD2-15 does not affect cell proliferation in untransformed, healthy cells, proliferation assays were performed on ZKS cells, primary kidney stromal cells derived from adult zebrafish. ZKS cells were exposed to 15–60 μM of NHD2-15 and imatinib and enumerated with a cell counter after 72 h of exposure (S1C Fig). In the presence of NHD2-15 cell growth still increased, and remained similar when compared to ZKS cells treated with imatinib (S1C Fig), indicating that NHD2-15 did not inhibit the proliferation of these healthy primary cells.

### NHD2-15 antagonist toxicity

To determine if NHD2-15 was toxic to cells and living organisms, we performed an adult zebrafish toxicity assay. Zebrafish are useful for these studies, as our compounds are water soluble. Additionally, zebrafish are an inexpensive, efficient, whole-organism model for drug screening and validation [42–45]. Fish were placed into a tank and 15 μM of antagonists were added; 12 fish were analyzed every 4 h for three days to assess their health. Exposure to DMSO (8 fish) and imatinib (8 fish) had no effect on fish health or survival (S2A Fig). NHD2-15 also had no negative effects and was non-toxic at 15 μM; all fish were healthy and survived the experiment (S2A Fig). However, increasing the dose to 30 μM killed all fish (8 fish) over the course of 6 hours (S2B Fig). Due to the fact that all fish survived 30 μM for at least 2 hours, we performed another experiment whereby four fish were exposed to 30 μM for 2 hours, and then placed into fresh water. All fish survived this treatment (S2C Fig). These findings indicate that while NHD2-15 prevents proliferation of leukemic cells, it is also non-toxic to zebrafish over 72 h of exposure at 15 μM. It also indicates that increasing the concentration of NHD2-15 is tolerable, but only for a short amount of time.

### NHD2-15 prevents activation of BCR-ABL1

**Fig 6:**
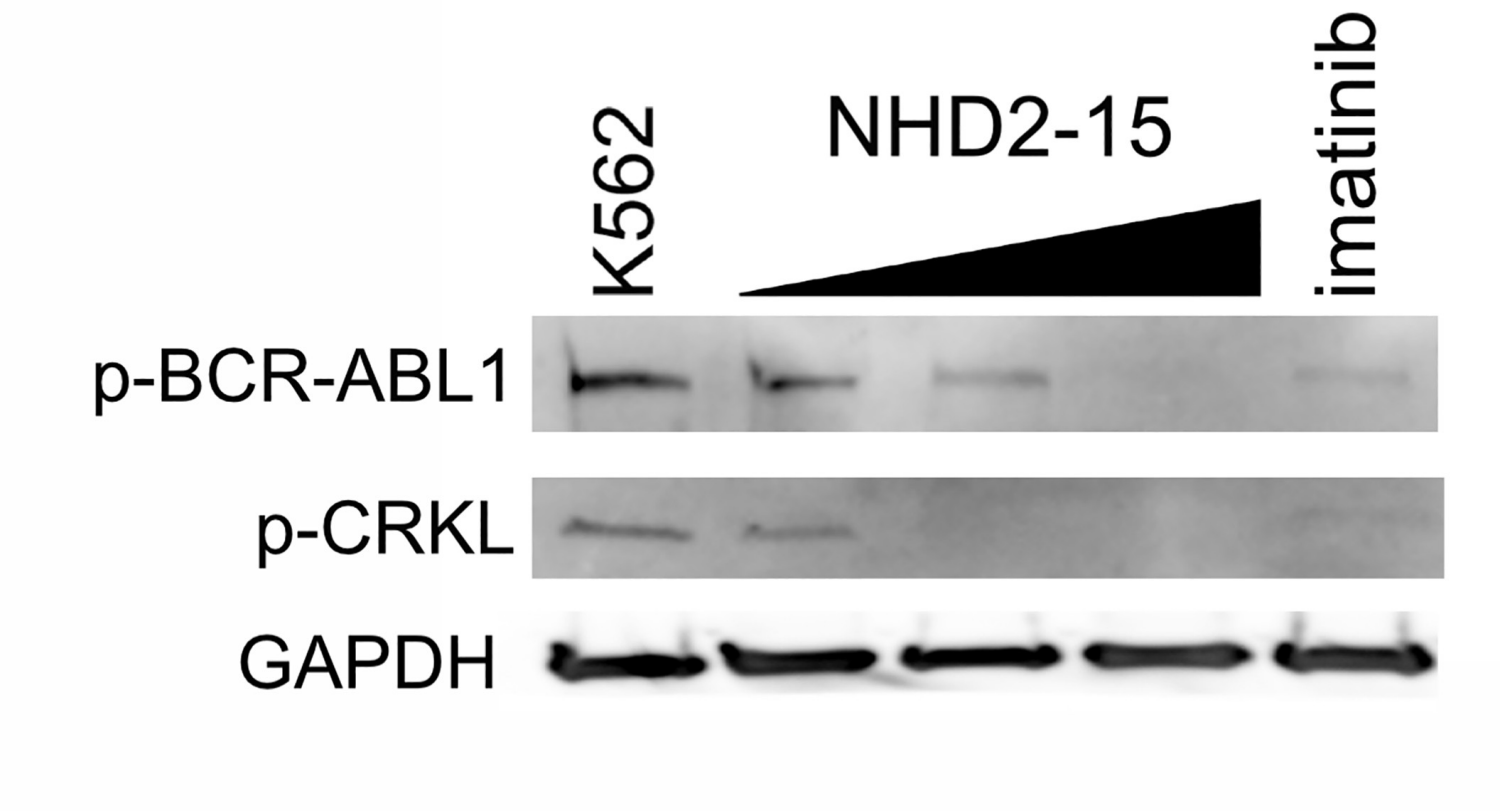
NHD2-15 reduces phosphorylation of BCR-ABL1 and CRKL in a dose-dependent manner. K562 cells were treated with DMSO vehicle (K562, left lane), increasing levels of NHD2-15 (black triangle spans 15, 30, and 60 μM), and 1 μM imatinib (right lane) for 72 hours. 10 μg of total cell lysate per lane was probed for phosphorylated (p) BCR-ABL1 and CRKL. GAPDH is shown below as loading control.

To determine if NHD2-15 prevented the activation of cell proliferation pathways upregulated in leukemogenesis, K562 cells were treated with NHD2-15 for 72 h. A western blot for phospho-BCR-ABL1 and phospho-CRKL, a downstream adapter protein that functions as a prognostic indicator in patients with CML [46], was performed. Increasing amounts of NHD2-15 caused decreased activation of BCR-ABL1 and CRKL in a similar manner to 1 μM imatinib (Fig 6). GAPDH (Fig 6**)** was used as a loading control. These data indicated that activated (phospho) BCR-ABL1 and CRKL were reduced by NHD2-15 addition, explaining the reduction in leukemic proliferation.

### Combinatorial addition of NHD2-15 and imatinib kills K562 cells more efficiently

**Fig 7:**
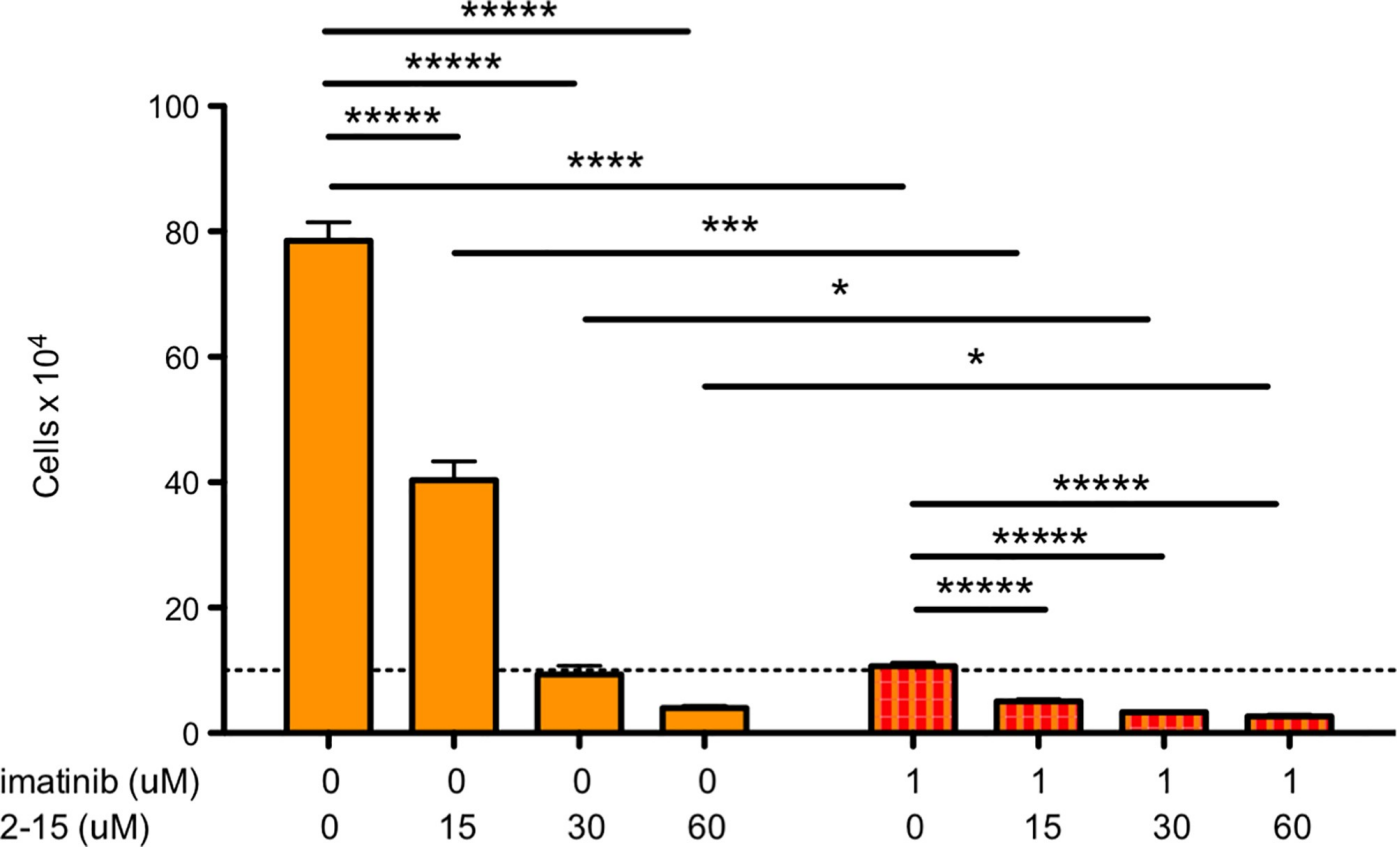
Combinatorial addition of NHD2-15 and imatinib reduces proliferation of K562 cells. 1×10^5^ K652 cells were incubated with 0, 15, 30, or 60 μM of NHD2-15 only (orange bars) and with 1 μM of imatinib (red and orange striped bars). After 72 hours, cells were enumerated by trypan blue exclusion. Dashed line denotes starting amount of cells. Bars represent the mean, and error bars represent SD; * p = 0.04, ** p = 0.03, *** p = 0.006; **** p = 0.001, ***** p < 0.001; n = 3 for K652 samples and n = 4 for combinatorial samples.

Finally, we hypothesized that NHD2-15 and imatinib added combinatorially would significantly reduce K562 cell proliferation. To test this, we added NHD2-15 and imatinib together and performed a cell proliferation assay. After K562 cells were exposed to 15–60 μM of NHD2-15 and 1μM of imatinib for 72 h they were enumerated with a cell counter. As the concentration of NHD2-15 increased, cell growth was significantly reduced (Fig 7) indicating that the combination of NHD2-15 with imatinib significantly killed K562 cells better when compared to cells exposed to imatinib alone.

## Discussion

In this study, we generated four novel antagonists of GRB2. SPR data was collected to assess binding of these antagonists to GRB2, but since the data was not optimal for all compounds, we decided to more closely examine NHD2-15, which showed the most potent binding. NHD2-15 bound specifically to the SH2 domains of GRB2 when analyzed by a CN binding assay. NHD2-15 also reduced the amount of cellular proliferation in K562 cells when measured by metabolic and cell proliferation assays. NHD2-15 appears to be specific for cells overexpressing GRB2, as it didn’t kill primary cell lines, PBMCs, or adult zebrafish at low concentrations. It also appears to down regulate the same proteins as imatinib, and works even better at killing cells when combined with imatinib. For these reasons, we postulate that NHD2-15 could be an effective molecule to treat BCR-ABL1^+^ leukemia.

Design of the furo-quinoxaline scaffold of our novel antagonists was inspired by a previously studied SH2 domain-binder of a GRB protein [47] (S3 Fig). This compound has a central quinoxaline, and computational modeling revealed that the 2-hydroxyl and α-hydrogen off the 3-position of the quinoxaline adopt a planar conformation in the binding site. To increase the overall rigidity and ensure this planarity, we cyclized this central core of the compound, which led to our furo-quinoxaline scaffold. After that, novel synthetic methods were used to prepare our library of furo-quinoxaline compounds. Despite their utility as intermediates in chemical synthesis [48], and as rigid scaffolds for novel drug development [49, 50], fused furo-quinoxalines have not been intensively studied, likely due to the difficulty of a versatile method of synthesis. The separate works of Iijima and Ismail report the cyclization of hydroxyl-substituted quinoxalines by use of phosphorous oxychloride, but only a few examples were described by Iijima, and both methods only yield mono-substituted furo-quinoxalines as opposed to more versatile di-substituted furo-quinoxalines [49, 51]. Kumar and co-workers described the synthesis of a benzofuran fused with a quinoxaline scaffold by use of a strong Lewis acid, and successfully prepared a multitude of compounds by this method [52]. However, their synthesis only provided the benzofuran-fused heterocycle as opposed to a lone furan. Other work by Nakhi *et al*. detailed the cyclization of alkyne-substituted quinoxalines; this method is also limited to the formation of only mono-substituted furo-quinoxalines [50]. Additionally, this method consists of two discrete synthetic steps: installation of the alkyne followed by cyclization. Our novel method employed a domino S_RN_1 *C*-arylation of a *β*-diketone with 2,3-dichloroquinoxaline, followed by *O*-arylation via S_N_Ar to yield versatile di-substituted furo-quinoxalines in only one synthetic step.

Identifying the conditions for our key domino reaction took extensive optimization. Initial attempts to build the furo-quinoxaline scaffold were based on recent work by Aljaar *et al*. which utilized an intermolecular Ullmann-type *C*-arylation between 1,2-dihalobenzenes and *β*-dicarbonyls with subsequent Ullmann-type *O*-arylation to build benzofurans [53]. Disappointingly, application of this method with 2,3-dibromoquinoxaline and 2,4-pentanedione did not furnish the desired furo-quinoxaline and instead gave a complex mixture of aromatic compounds. Thus, efforts turned toward a possible tandem S_N_Ar carbon-coupling with a subsequent S_N_Ar of the resulting enolate. The *C*-arylation step was first investigated by forming the methylene anion of diethylmalonate with sodium hydride, followed by addition of 2,3-dichloroquinoxaline, and isolation of the desired coupled product suggested the feasibility of this approach. These same conditions were used to couple methyl acetoacetate with 2,3-dichloroquinoxaline, and although the *C*-arylated product could not be isolated, the desired cyclized product was isolated with 14% yield, again suggesting that a tandem S_N_Ar *C*-arylation and S_N_Ar *O*-arylation would be an effective method in preparing furo-quinoxalines. Unfortunately, despite a thorough screen of conditions, this isolated yield of 14% could not be improved. This then led to a survey of conditions to promote a domino reaction consisting of an S_RN_1 mediated carbon-coupling, followed by S_N_Ar with the resulting enolate. There have been a number of reports demonstrating the addition of ketone and *β*-dicarbonyl enolates to different heterocycles via S_RN_1 mechanisms [54], one of which even notes a single entry of an interesting cyclization similar to what is described here but in poor yield [55].

After exhaustive investigation of our novel S_RN_1/S_N_Ar domino reaction, our optimized conditions consisted of *β*-diketone addition directly to sodium hydride, followed by the addition of catalytic iron (II) sulfate and 2,3-dichloroquinoxaline. Reaction times of around 6 h yielded the most product; longer reaction times showed some decomposition of the product when the reaction was followed using GC-MS. Despite extensive optimization of these reaction conditions, this valuable transformation suffered from poor yields and a substrate scope limited to the compounds discussed here. Even subsequent functionalizations of the products, such as hydrolysis of the ester in NHD2-92 to a carboxylic acid, were unattainable. Nonetheless, our useful S_RN_1/S_N_Ar domino reaction enabled the efficient one-step preparation of our library compounds utilizing novel synthetic methods.

We used the BCR-ABL1^+^ human leukemic cell line K562 to assess if these compounds prevented cellular growth. We performed these studies on K562 cells for multiple reasons. First, K562 cells are easily grown and manipulated *in vitro*. Secondly, they overexpress GRB2, and GRB2 overexpression is correlated with CML. It is important to note that K562 cells likely overexpress GRB2 even more than our studies indicate; we compared levels to that of HEK293FT cells, which are not cancerous, but still transformed to grow indefinitely *in vitro*. While we originally tested the efficacy of the compounds with CellTiter 96 assays, we were concerned that this might not be the best method, as this measures cellular metabolism. While metabolism is linked to cell proliferation, we wanted to count cells and ensure that they were not proliferating. The findings in both assays look very similar; at higher concentrations, the CellTiter 96 assay indicated that NHD2-15 reduced the metabolic activity, and our cell counts indicated decreased proliferation of the K562 cells. While NHD2-15 did not actively kill the cells as potently as imatinib, it had a profound effect on halting their growth in these assays. Importantly, it had no effect on primary human PBMCs or primary zebrafish kidney cells, indicating that it is likely specific for blocking the proliferation of transformed cells.

One other issue that we addressed was toxicity. We performed these experiments by adding NHD2-15 to the water of adult zebrafish, as they are convenient animals for studying drug efficacy and toxicity in vertebrates [56, 57]. We hypothesized that NHD2-15 would be relatively non-toxic, as it was designed towards the SH2 domain of a specific protein overexpressed in cancer cells. To assess this, we added the lowest concentration of compounds that had a statistically significant negative effect on cellular proliferation in our *in vitro* assays (15 μM) to the water of adult zebrafish. We observed that NHD2-15 was nontoxic, with fish surviving over 3 days in 15 μM of the compound dissolved in aquarium water. Importantly, increasing the concentration of NHD2-15 did kill fish after only 6 h of exposure. However, the toxicity of NHD2-15 at 30 μM does not automatically preclude it from being an effective treatment for blocking GRB2 signalling; reduced exposure time of only 2 hours showed no toxicity. Importantly, delivery methods of this drug may also need to be optimized. Unfortunately, no good zebrafish models of GRB2^high^ myeloid leukemia exist. If developed, these diseased fish could be subjected to NHD2-15 to determine if it prevents or shrinks tumor burden in a live, whole animal system. It would also be useful to analyze if NHD2-15 has a negative effect on embryonic development, as GRB2 is expressed in dividing cells receiving RTK signals prevalent during embryogenesis and development.

GRB2 binds to BCR-ABL1 via a phosphorylated tyrosine residue on BCR with its SH2 domain. This binding to BCR-ABL1 initiates leukemic transformation [25]. It is known that imatinib reduces the amount of phospho-BCR-ABL1 in leukemic cells, reducing the activated protein, and hence, downstream leukemic transformation. In our assays NHD2-15 had the same effect, reducing the amount of activated BCR-ABL1 present in K562 cells. Additionally, phospho-CRKL, an adapter protein that is a prognostic indicator of leukemic transformation [30, 46] (and a direct target of BCR-ABL1 [30, 58]) was also reduced with NHD2-15 and imatinib. These data indicate that NHD2-15 and imatinib both reduce essential pathways for leukemic transformation in K562 cells.

While NHD2-15 1) binds to GRB2 in the micromolar range, 2) binds the SH2 domain of GRB2, 3) down-regulates leukemic cell proliferation proteins, 4) and is non-toxic to non-transformed cells and in an animal model at 15 μM, it does not appreciably kill BCR-ABL1^+^ cells *in vitro;* it only prevents their proliferation. However, this is an important finding. Without NHD2-15 added to K562 cells, they increase 9-fold over the course of 72 h. When NHD2-15 is present, this expansion is completely blocked. In essence, NHD2-15 effectively prevents the proliferation of leukemic cells. In addition, our findings indicate that NHD2-15 is useful as an additive therapy for preventing the expansion of cancerous cells. When imatinib and NHD2-15 were simultaneously added to K562 cells, leukemic cell death was observed. By adding the antagonists simultaneously, cell proliferation was inhibited better than NHD2-15 or imatinib alone, indicating that NHD2-15 could potentially be used in situations where cancer cells have already become resistant to frontline TKI treatments. In essence, these studies indicate that NHD2-15 may have some therapeutic efficacy in treating CML, albeit through a different mechanism than current TKIs. Future studies focused on analyzing this possibility should be fruitful, helping to prevent deaths attributed to this hematologic malignancy.

## Supporting information

S1 Fig Treatment with NHD2-15 does not kill primary cell lines. (A) 1×10^5^ primary human PBMCs cells were incubated with increasing amounts of NHD2-15. Bars represent the mean, and error bars represent SD. N.S., not significant (p values of 0.139, 0.203, and 0.219); n = 3 for all trials. Cells were also cultured with 1 μM imatinib (B). After 72 h, cells were enumerated by trypan blue exclusion. Dashed line denotes starting amount of cells. Bars represent the mean, and error bars represent SD. N.S., not significant (p = 0.72); n = 4 for all trials. (C) 1×10^5^ primary ZKS cells were incubated with 0, 15, 30, or 60 μM of NHD2-15. Bars represent the mean, and error bars represent SD. N.S., not significant (p values of 0.880, 0.537, and 0.746); n = 4 for all trials. Cells were also cultured with 1 μM of imatinib (D). After 72 hours, cells were enumerated by trypan blue exclusion. Dashed line denotes starting amount of cells. Bars represent the mean, and error bars represent SD; N.S., not significant (p = 0.877); n = 4 for all trials.
(PPTX)

S2 Fig NHD2-15 at 15 μM is not toxic to adult zebrafish. (A) 6-month-old zebrafish were placed in water containing 15 μM GRB2 antagonist (orange line), 2μM imatinib (red line), or vehicle control (DMSO, black line) and monitored over 3 days for survival. (B) 6-month-old zebrafish were placed in water containing 30 μM GRB2 antagonist (orange line) and monitored for 6 h. (C) 6-month-old zebrafish were placed in water containing 30 μM GRB2 antagonist (orange line) for 2h, then moved to fresh water and monitored over 3 days for survival.
(PPTX)

S3 Fig Compound library design. Structure of a previously studied GRB SH2 domain-binder [47] and depiction of the rational design of our library compounds.
(PPTX)

S4 Fig Raw SPR sensorgrams for NHD2-15 versus GRB2. Concentrations (From the top): 125, 62.5, 31.25, 15.625, 7.8125, 3.90625, 0 μM in HBSEP buffer with 0.5% DMSO.
(PPTX)

S5 Fig Dose-response curve resulting from SPR data of NHD2-15 versus GRB2. Affinity: *K*_D_ = 119 ± 2 μM as determined using Scrubber 2.0 software.
(PPTX)

S6 Fig Raw SPR sensorgrams for NHD2-92 versus GRB2. Concentrations (From the top): 125, 62.5, 31.25, 0 μM in HBSEP buffer with 0.5% DMSO.
(PPTX)

S7 Fig Dose-response curve resulting from SPR data of NHD2-92 versus GRB2. Affinity: *K*_D_ = 1000 ± 20 μM as determined using Scrubber 2.0 software.
(PPTX)

S8 Fig Raw SPR sensorgrams for NHD2-107 versus GRB2. Concentrations (From the top): 125, 62.5, 0 μM in HBSEP buffer with 0.5% DMSO.
(PPTX)

S9 Fig Dose-response curve resulting from SPR data of NHD2-107 versus GRB2. Affinity: *K*_D_ = 3400 ± 100 μM as determined using Scrubber 2.0 software.
(PPTX)

S10 Fig Raw SPR sensorgrams for NHD2-114 versus GRB2. Concentrations (From the top): 125, 62.5, 31.25, 15.625, 0 μM in HBSEP buffer with 0.5% DMSO.
(PPTX)

S11 Fig Dose-response curve resulting from SPR data of NHD2-114 versus GRB2. Affinity: *K*_D_ = 440 ± 7 μM as determined using Scrubber 2.0 software.
(PPTX)

S12 Fig Original blot for Fig 4. (PPTX)

S13 Fig Original blots for Fig 6. (PPTX)

S1 Data Lewis supporting information raw data. (XLSX)

S1 Table Equilibrium dissociation constants (*K*_D_) values as measured by SPR, which were used to assess binding affinity between compounds and the GRB2 protein. (PPTX)
