# Supplementary figures and images for: NHD2-15, a novel antagonist of Growth Factor Receptor-Bound Protein-2 (GRB2), inhibits leukemic proliferation
Source: PLoS One. 2020 Aug 11;15(8):e0236839. doi: 10.1371/journal.pone.0236839 (PMC7418987; doi:10.1371/journal.pone.0236839)

## Slide 1
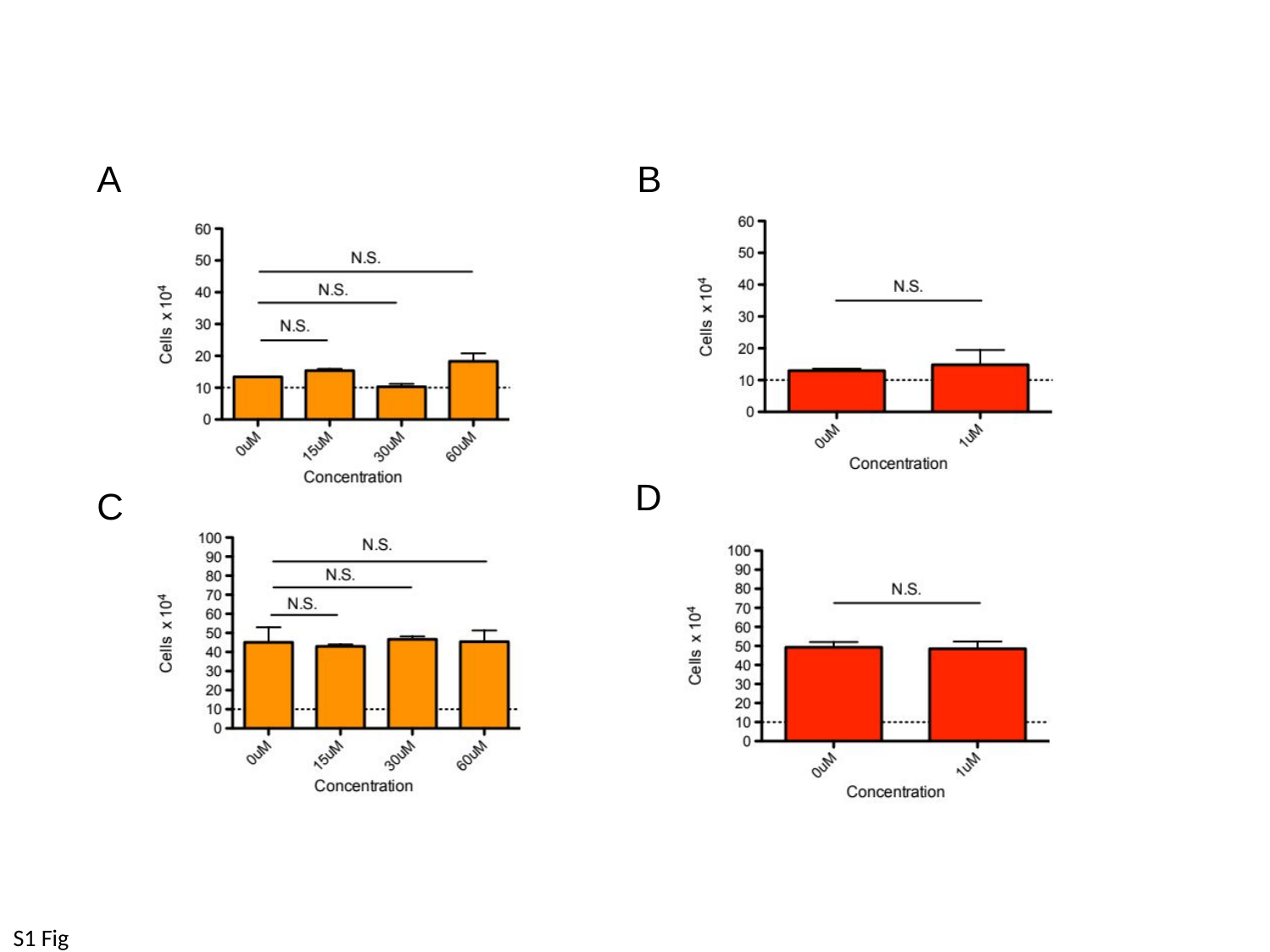

B
A
D
C
S1 Fig

Supplement: S1 Fig — (A) 1×10^5^ primary human PBMCs cells were incubated with increasing amounts of NHD2-15. Bars represent the mean, and error bars represent SD. N.S., not significant (p values of 0.139, 0.203, and 0.219); n = 3 for all trials. Cells were also cultured with 1 μM imatinib (B). After 72 h, cells were enumerated by trypan blue exclusion. Dashed line denotes starting amount of cells. Bars represent the mean, and error bars represent SD. N.S., not significant (p = 0.72); n = 4 for all trials. (C) 1×10^5^ primary ZKS cells were incubated with 0, 15, 30, or 60 μM of NHD2-15. Bars represent the mean, and error bars represent SD. N.S., not significant (p values of 0.880, 0.537, and 0.746); n = 4 for all trials. Cells were also cultured with 1 μM of imatinib (D). After 72 hours, cells were enumerated by trypan blue exclusion. Dashed line denotes starting amount of cells. Bars represent the mean, and error bars represent SD; N.S., not significant (p = 0.877); n = 4 for all trials. (PPTX) [file pone.0236839.s001.pptx]

## Slide 1
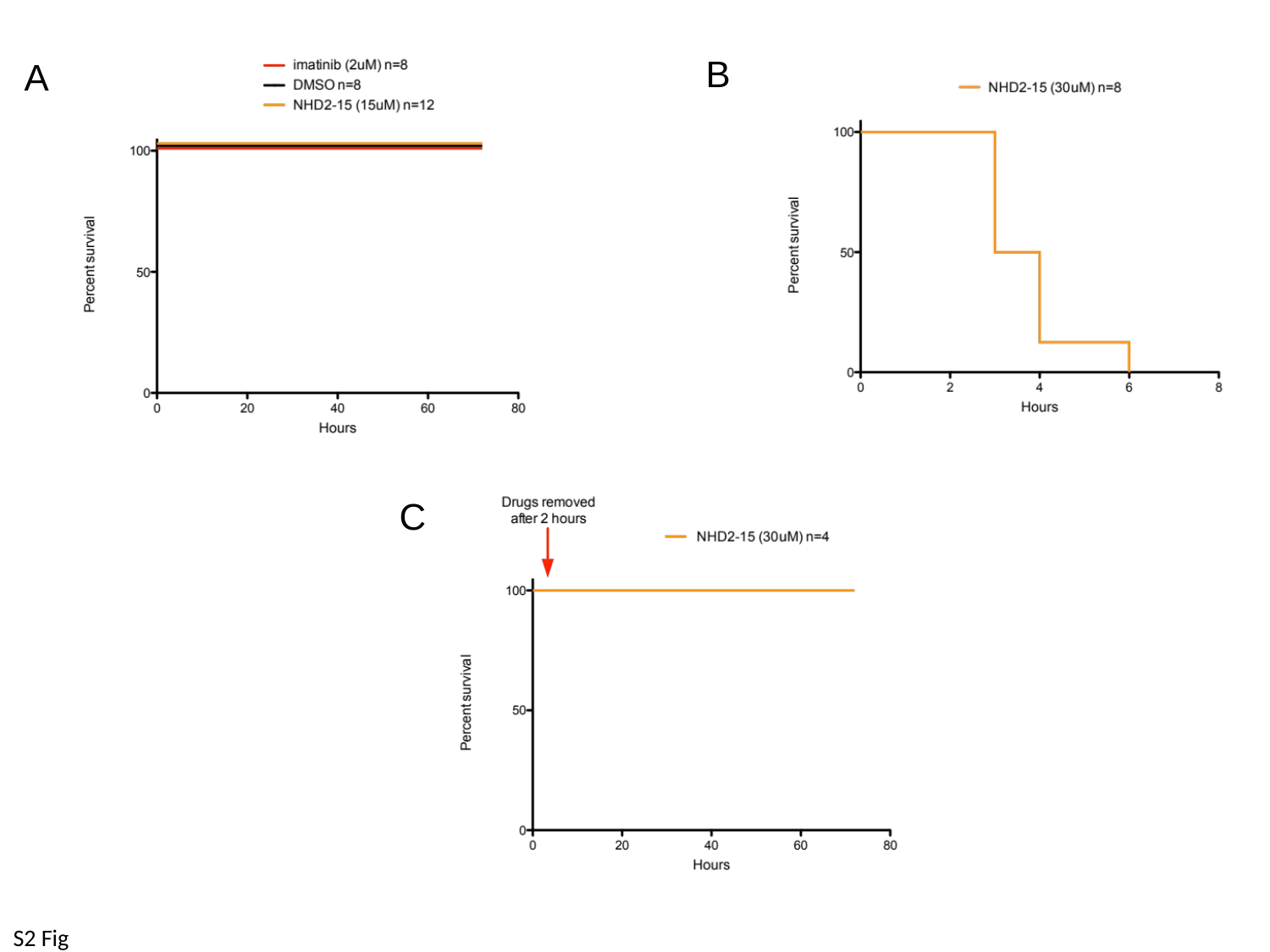

B
A
C
S2 Fig

Supplement: S2 Fig — (A) 6-month-old zebrafish were placed in water containing 15 μM GRB2 antagonist (orange line), 2μM imatinib (red line), or vehicle control (DMSO, black line) and monitored over 3 days for survival. (B) 6-month-old zebrafish were placed in water containing 30 μM GRB2 antagonist (orange line) and monitored for 6 h. (C) 6-month-old zebrafish were placed in water containing 30 μM GRB2 antagonist (orange line) for 2h, then moved to fresh water and monitored over 3 days for survival. (PPTX) [file pone.0236839.s002.pptx]

## Slide 1
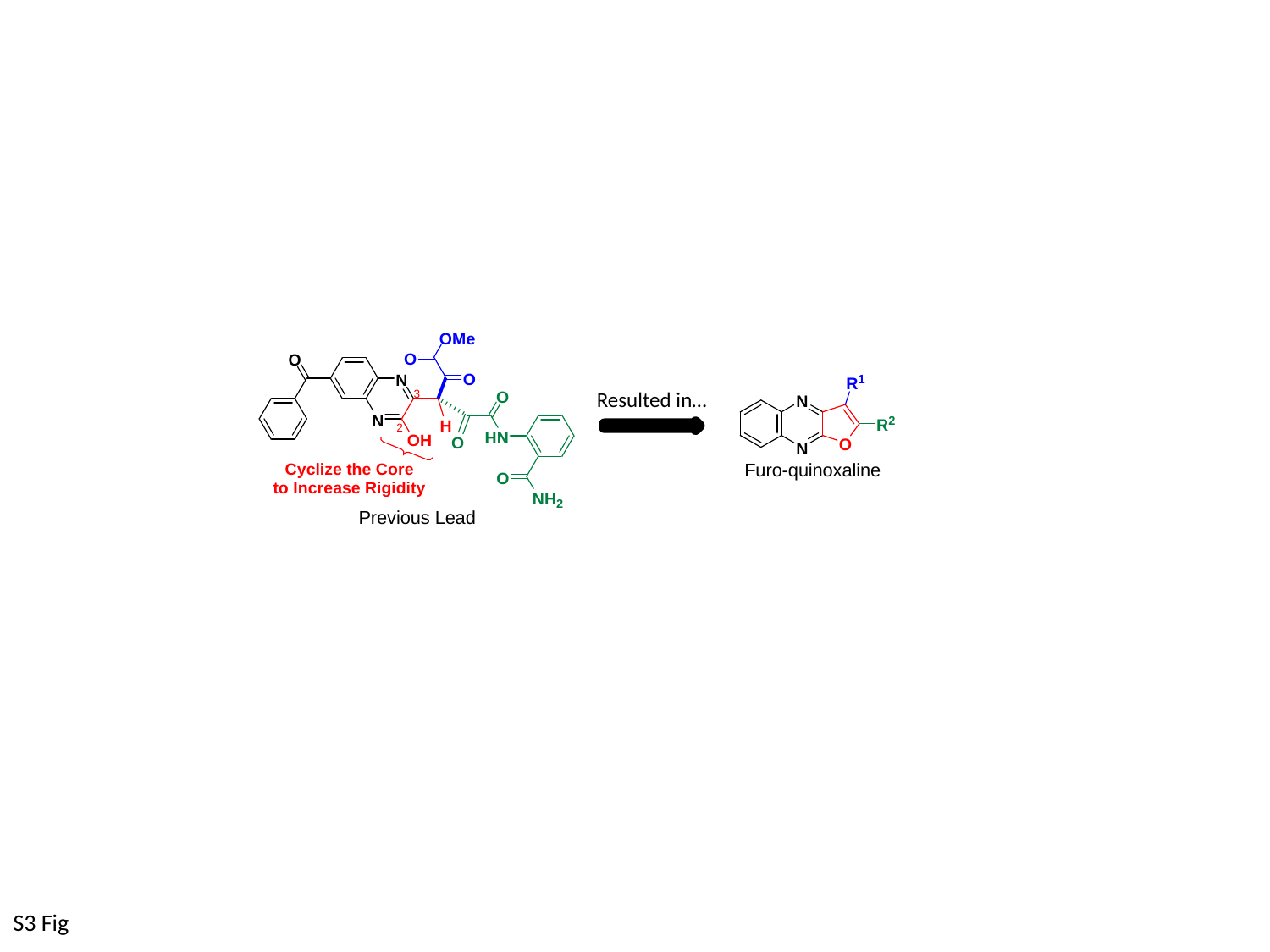

Resulted in…
Furo-quinoxaline
Previous Lead
S3 Fig

Supplement: S3 Fig — Structure of a previously studied GRB SH2 domain-binder [47] and depiction of the rational design of our library compounds. (PPTX) [file pone.0236839.s003.pptx]

## Slide 1
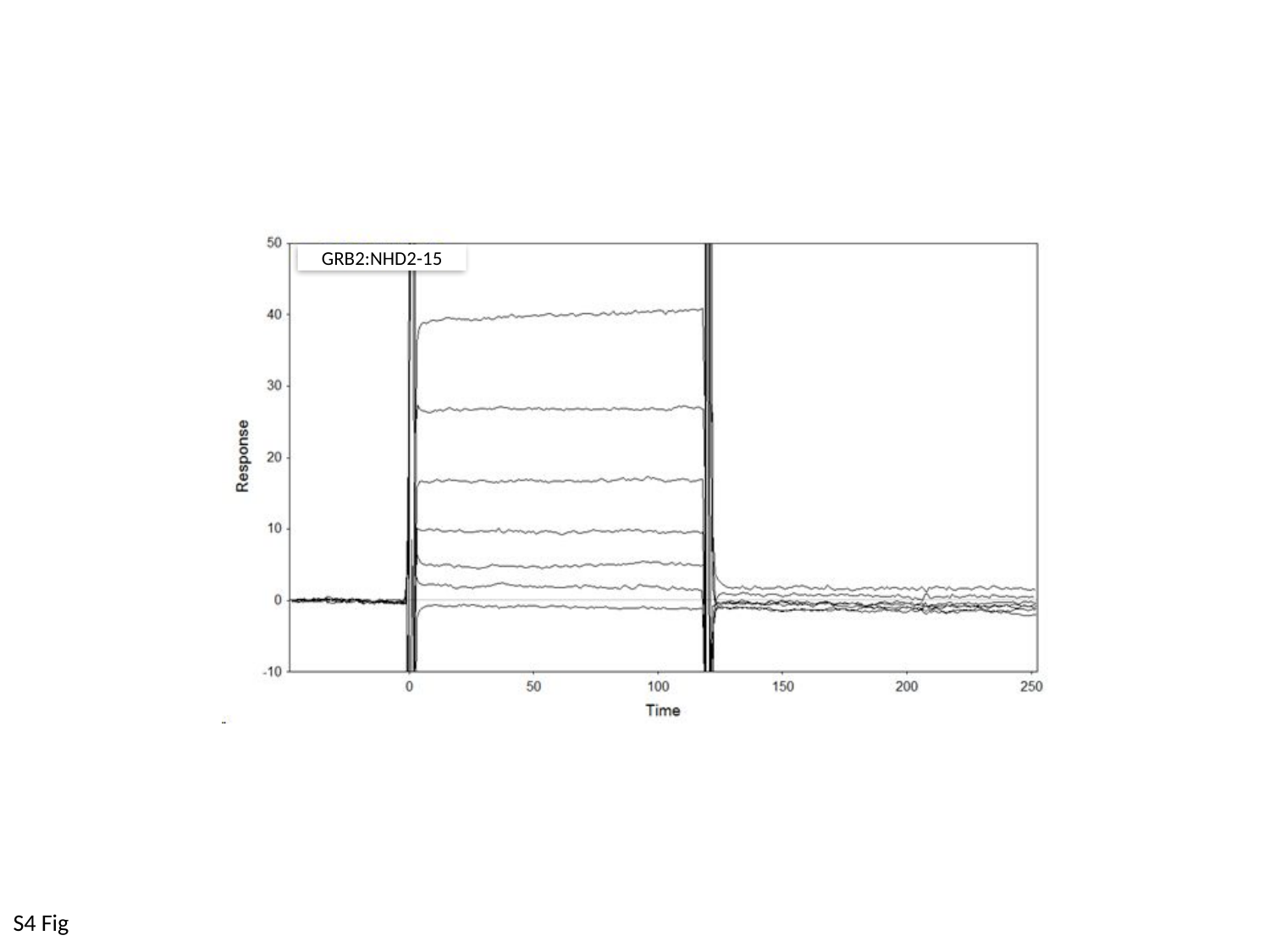

GRB2:NHD2-15
S4 Fig

Supplement: S4 Fig — Concentrations (From the top): 125, 62.5, 31.25, 15.625, 7.8125, 3.90625, 0 μM in HBSEP buffer with 0.5% DMSO. (PPTX) [file pone.0236839.s004.pptx]

## Slide 1
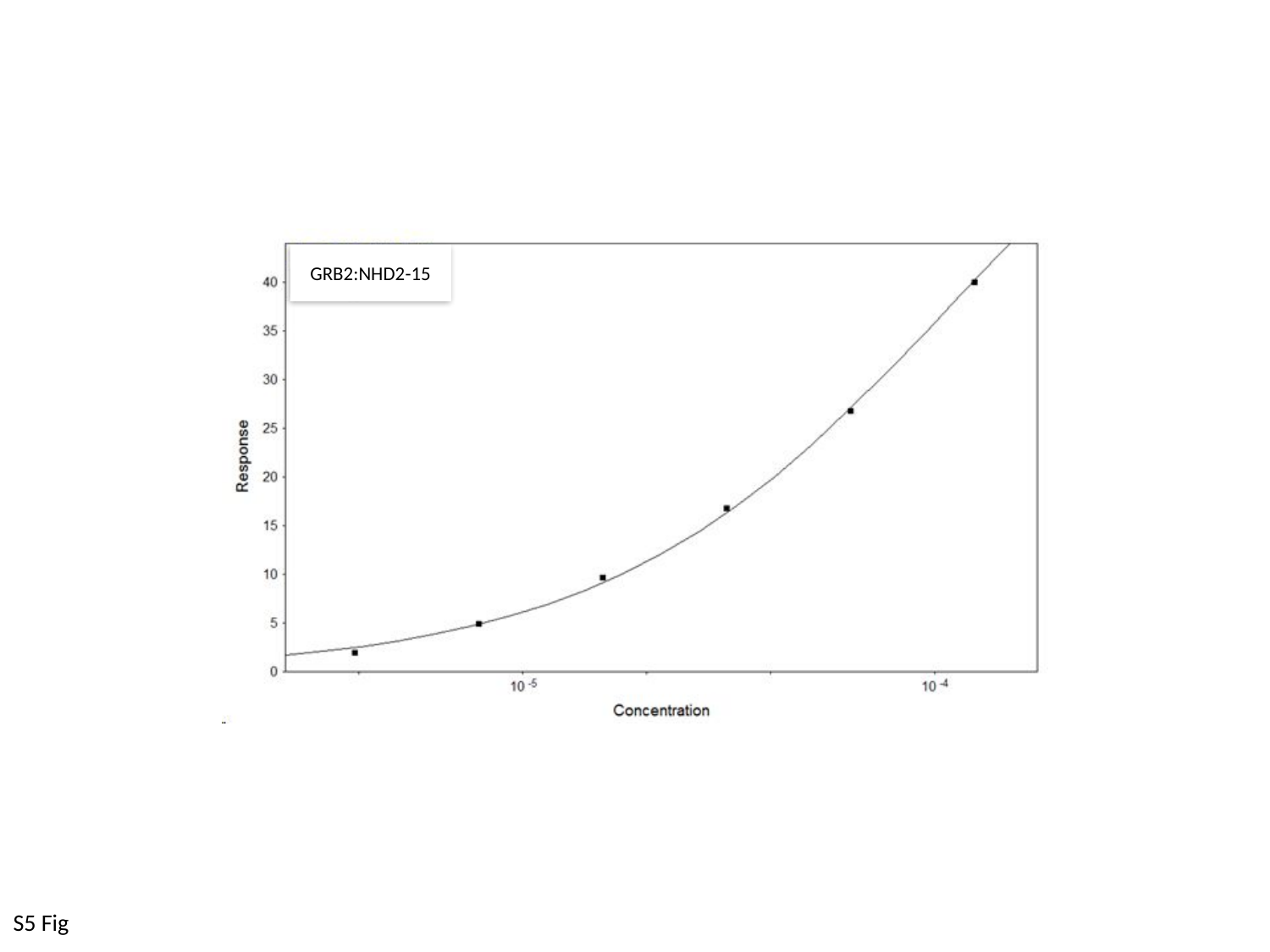

GRB2:NHD2-15
S5 Fig

Supplement: S5 Fig — Affinity: KD = 119 ± 2 μM as determined using Scrubber 2.0 software. (PPTX) [file pone.0236839.s005.pptx]

## Slide 1
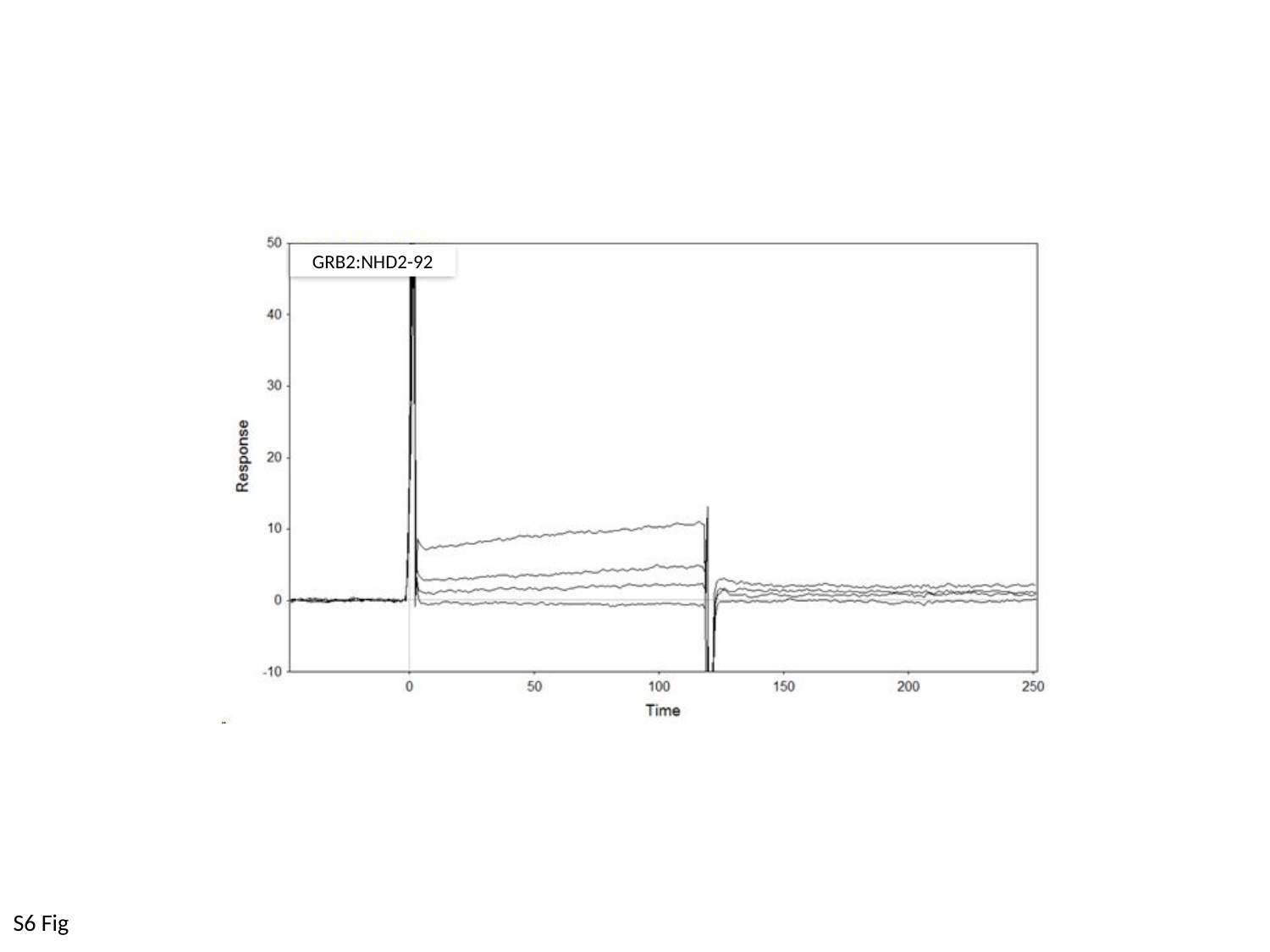

GRB2:NHD2-92
S6 Fig

Supplement: S6 Fig — Concentrations (From the top): 125, 62.5, 31.25, 0 μM in HBSEP buffer with 0.5% DMSO. (PPTX) [file pone.0236839.s006.pptx]

## Slide 1
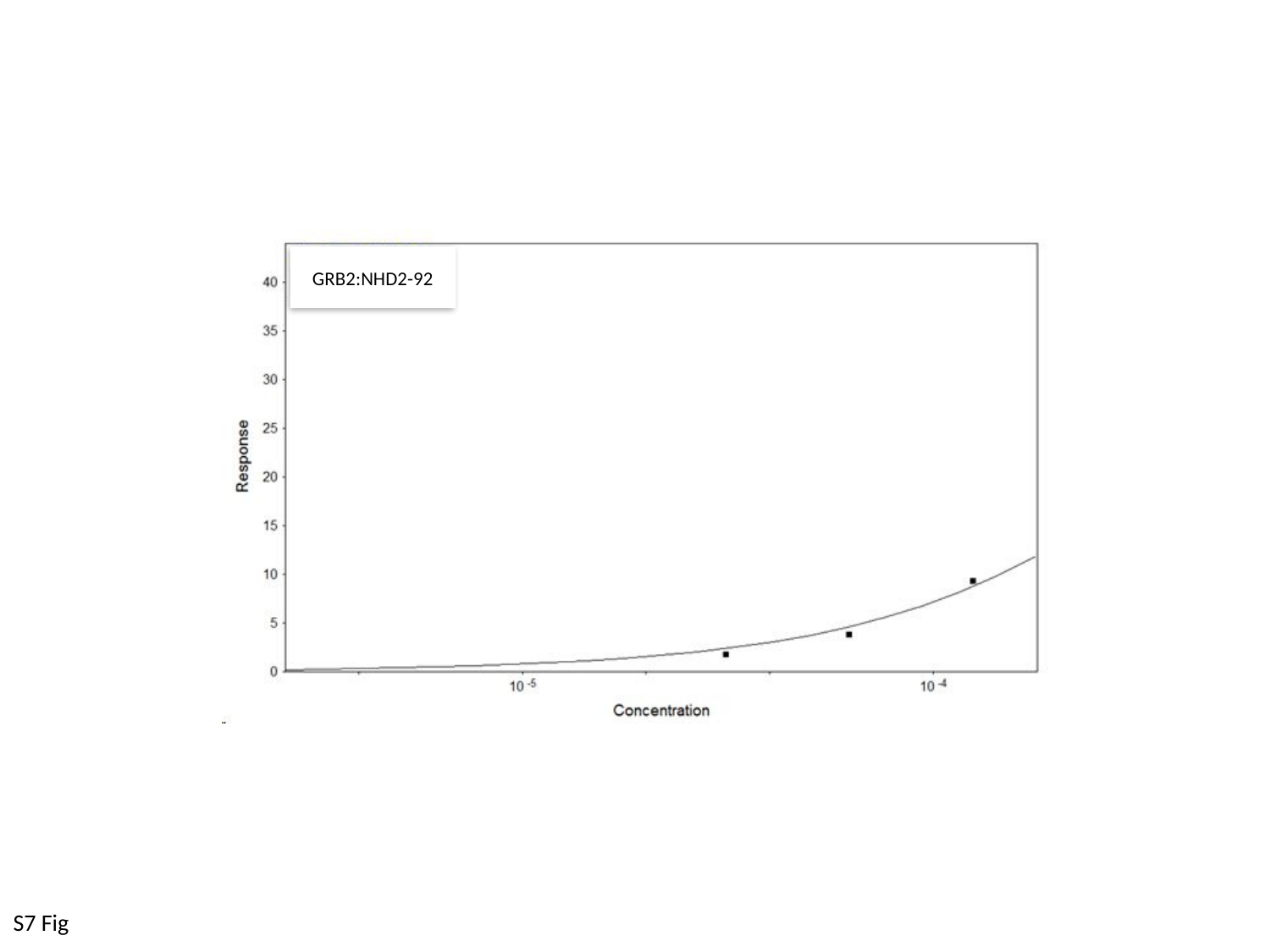

GRB2:NHD2-92
S7 Fig

Supplement: S7 Fig — Affinity: KD = 1000 ± 20 μM as determined using Scrubber 2.0 software. (PPTX) [file pone.0236839.s007.pptx]

## Slide 1
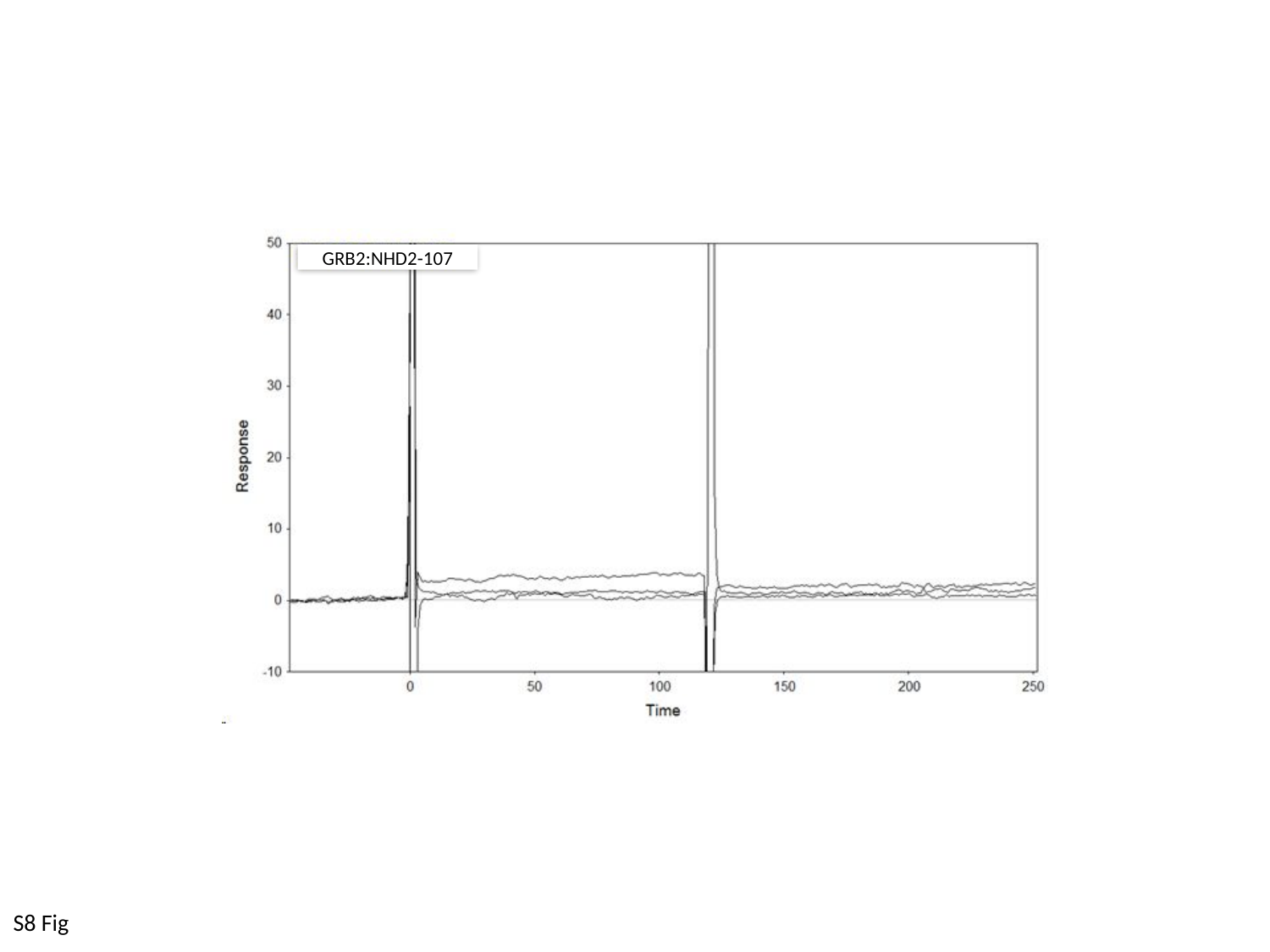

GRB2:NHD2-107
S8 Fig

Supplement: S8 Fig — Concentrations (From the top): 125, 62.5, 0 μM in HBSEP buffer with 0.5% DMSO. (PPTX) [file pone.0236839.s008.pptx]

## Slide 1
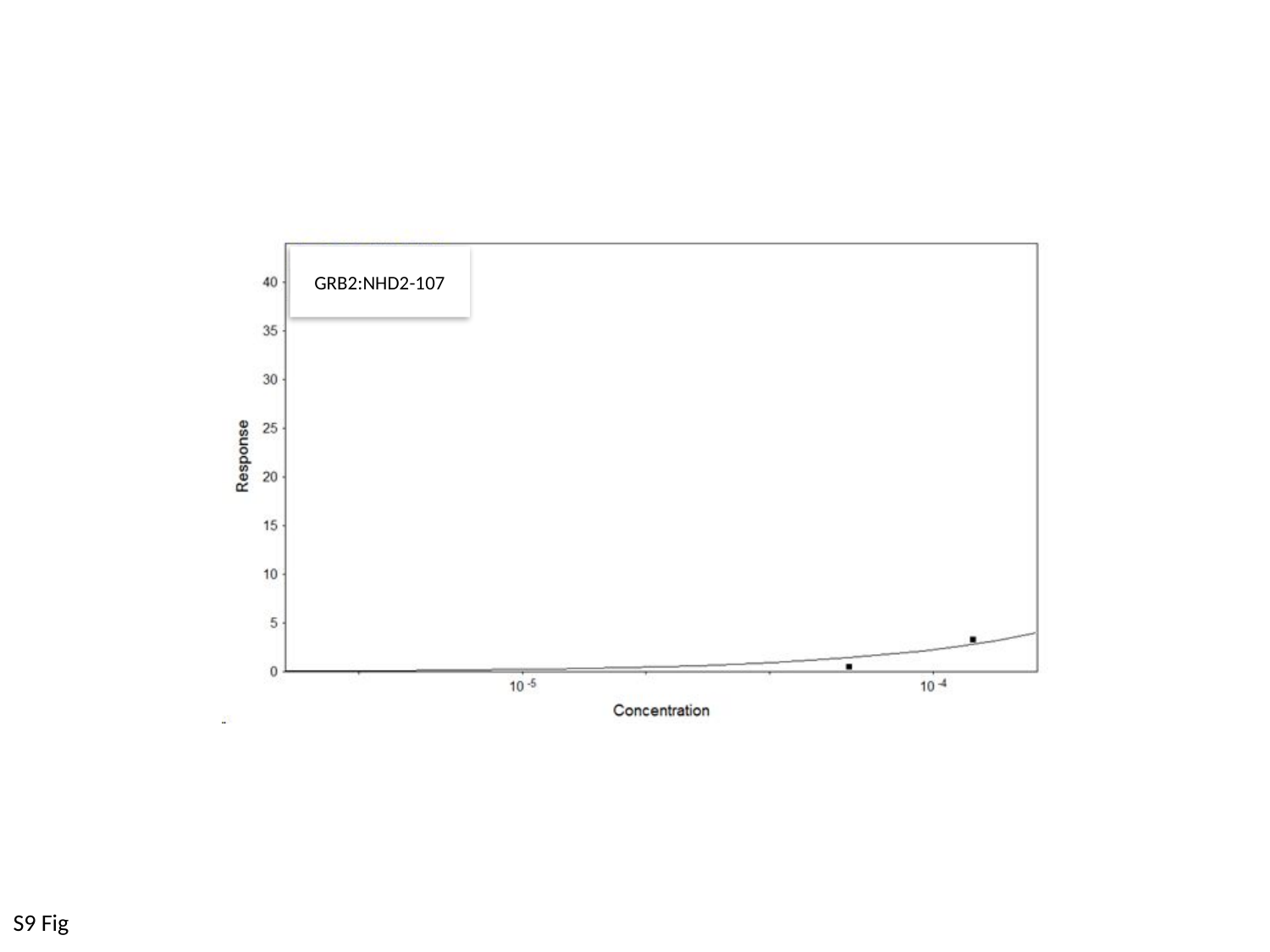

GRB2:NHD2-107
S9 Fig

Supplement: S9 Fig — Affinity: KD = 3400 ± 100 μM as determined using Scrubber 2.0 software. (PPTX) [file pone.0236839.s009.pptx]

## Slide 1
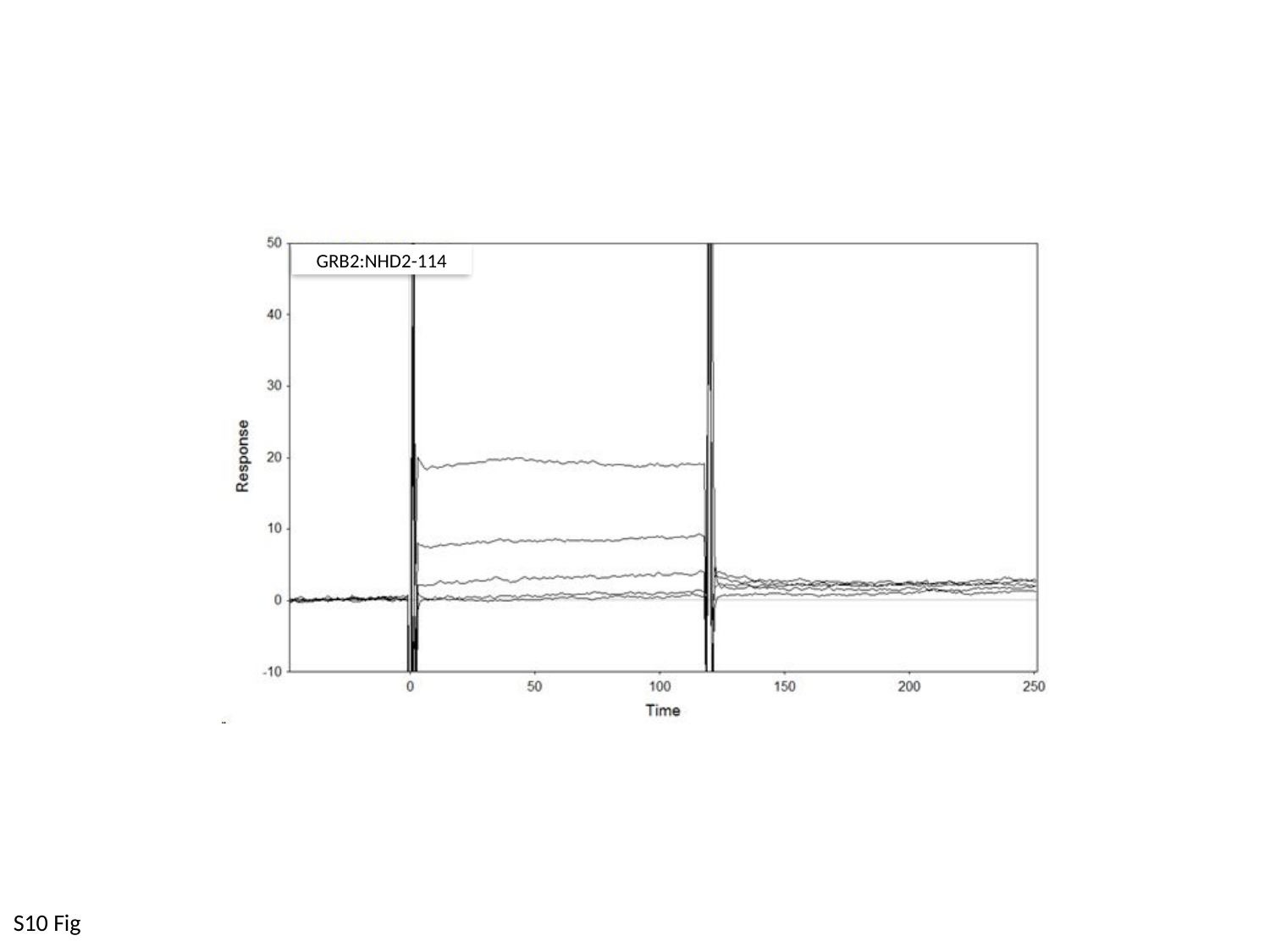

GRB2:NHD2-114
S10 Fig

Supplement: S10 Fig — Concentrations (From the top): 125, 62.5, 31.25, 15.625, 0 μM in HBSEP buffer with 0.5% DMSO. (PPTX) [file pone.0236839.s010.pptx]

## Slide 1
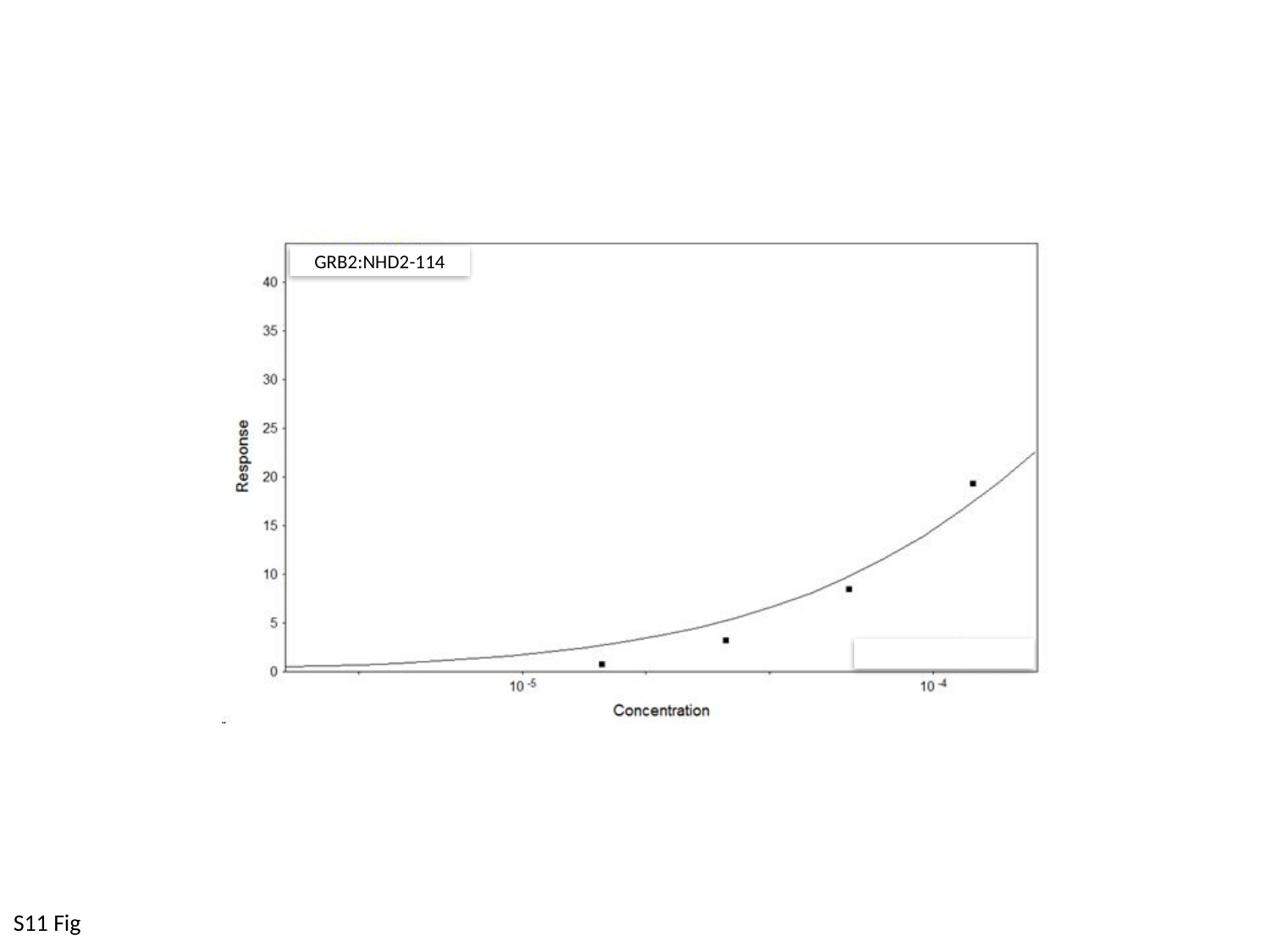

GRB2:NHD2-114
S11 Fig

Supplement: S11 Fig — Affinity: KD = 440 ± 7 μM as determined using Scrubber 2.0 software. (PPTX) [file pone.0236839.s011.pptx]

## Slide 1
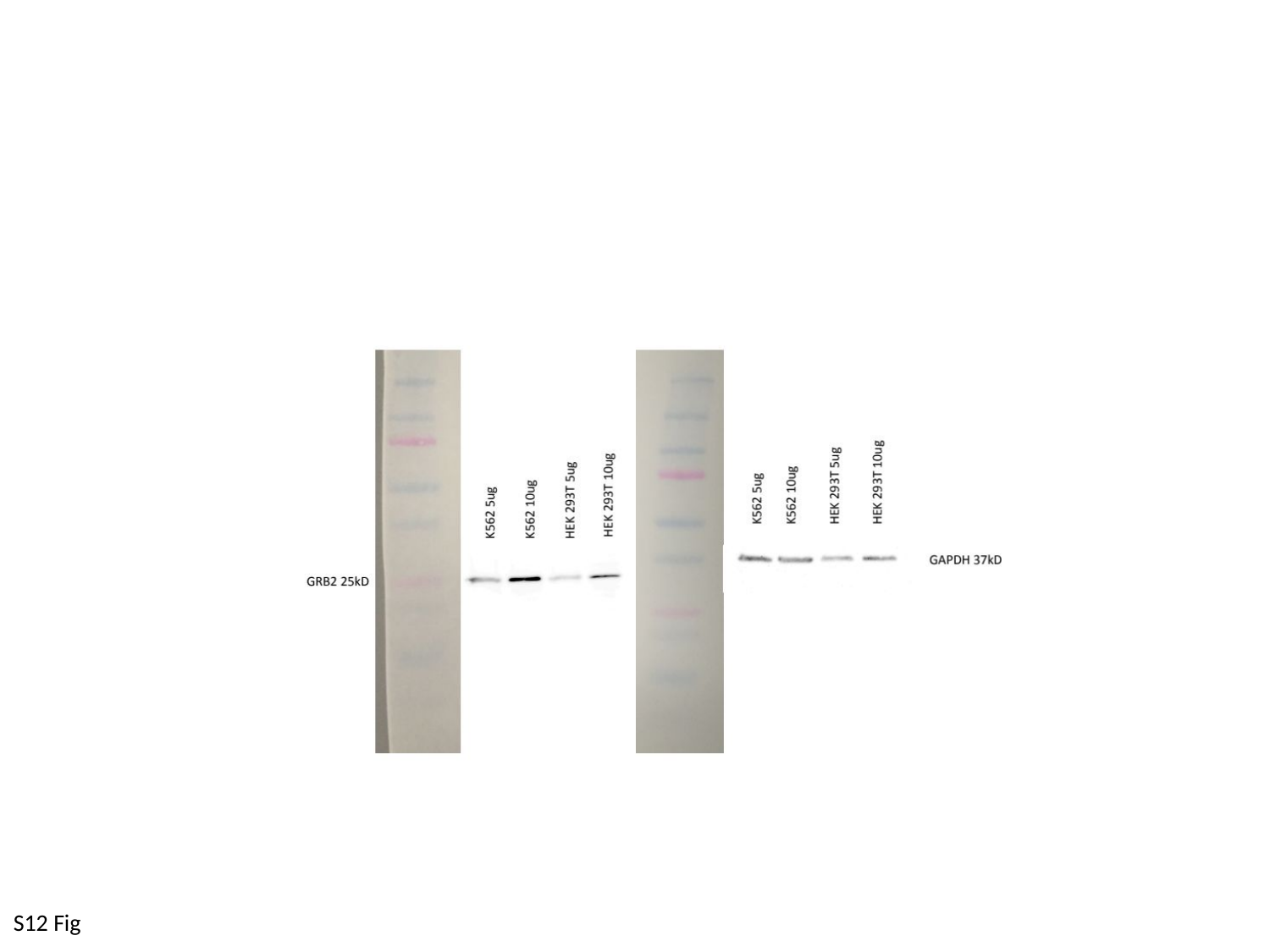

S12 Fig

Supplement: S12 Fig — (PPTX) [file pone.0236839.s012.pptx]

## Slide 1
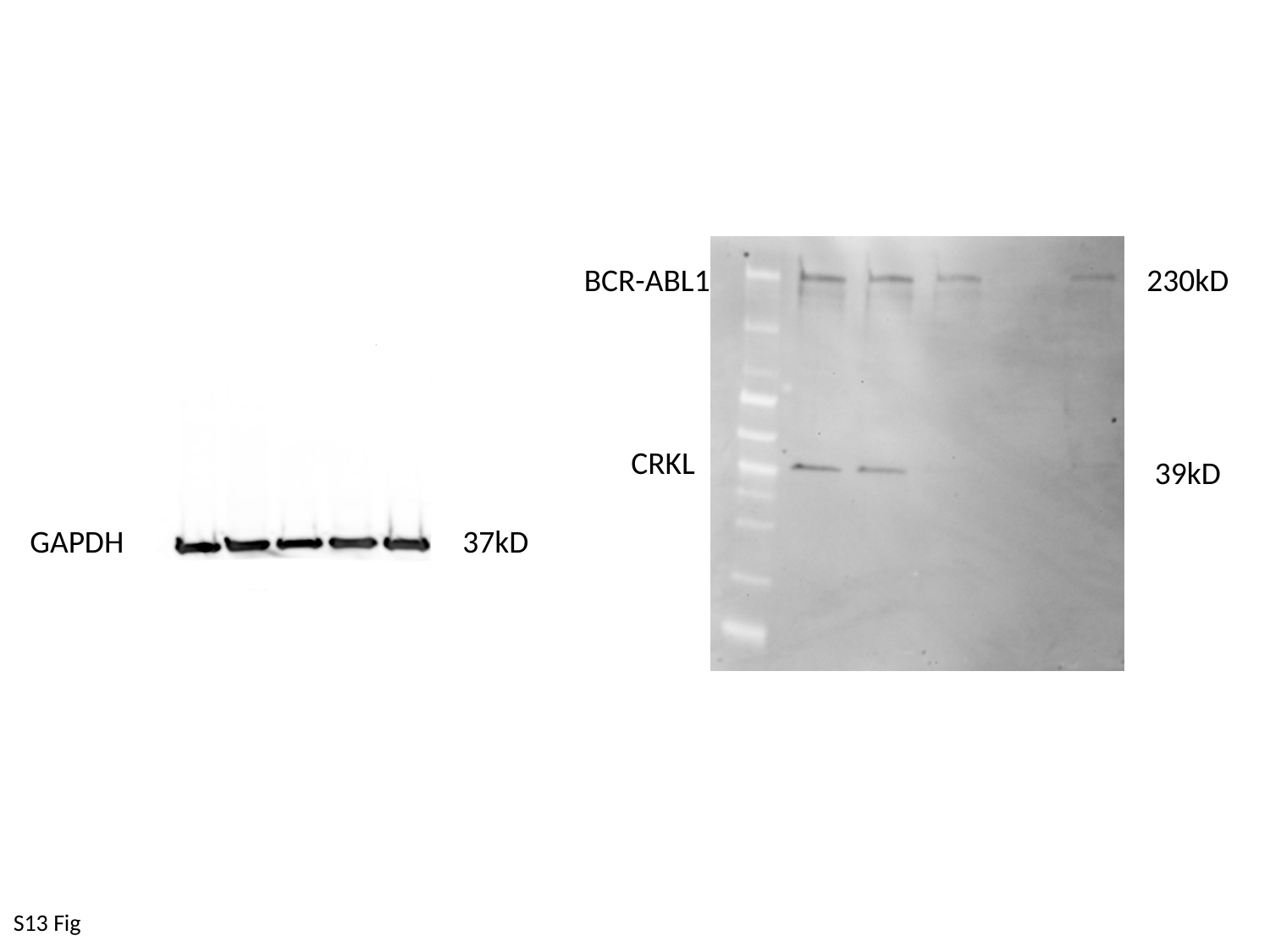

BCR-ABL1
230kD
CRKL
39kD
GAPDH
37kD
S13 Fig

Supplement: S13 Fig — (PPTX) [file pone.0236839.s013.pptx]
