# Supplementary material for: NHD2-15, a novel antagonist of Growth Factor Receptor-Bound Protein-2 (GRB2), inhibits leukemic proliferation
Source: PLoS One. 2020 Aug 11;15(8):e0236839. doi: 10.1371/journal.pone.0236839 (PMC7418987; doi:10.1371/journal.pone.0236839)
Supplement: S1 Table — (PPTX) [file pone.0236839.s015.pptx]

## Slide 1
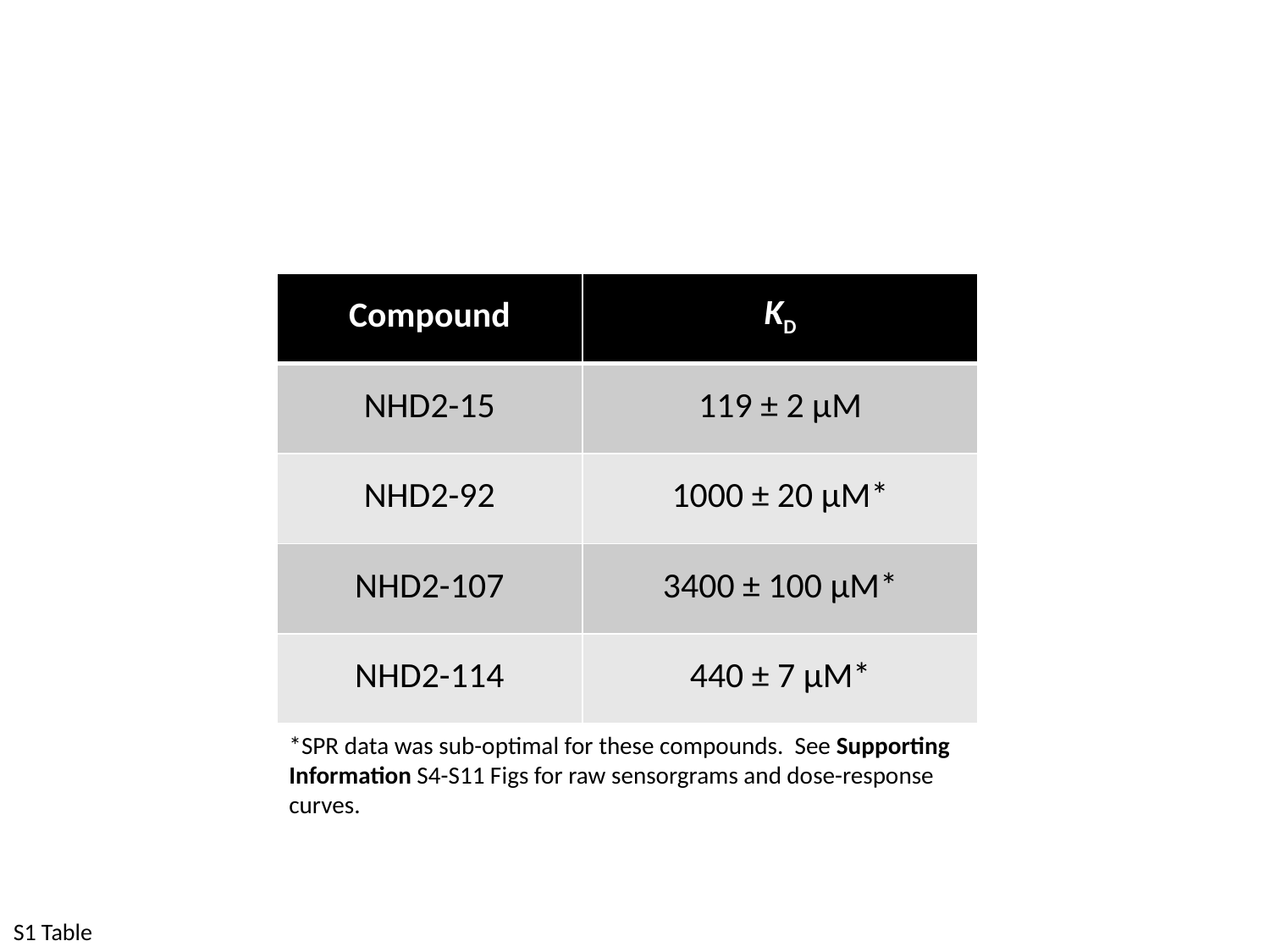

| Compound | KD |
| --- | --- |
| NHD2-15 | 119 ± 2 µM |
| NHD2-92 | 1000 ± 20 µM\* |
| NHD2-107 | 3400 ± 100 µM\* |
| NHD2-114 | 440 ± 7 µM\* |
*SPR data was sub-optimal for these compounds. See Supporting Information S4-S11 Figs for raw sensorgrams and dose-response curves.
S1 Table
