# Supplementary material for: NHD2-15, a novel antagonist of Growth Factor Receptor-Bound Protein-2 (GRB2), inhibits leukemic proliferation
Source: PLoS One. 2020 Aug 11;15(8):e0236839. doi: 10.1371/journal.pone.0236839 (PMC7418987; doi:10.1371/journal.pone.0236839)
Supplement: Attachment — Submitted filename: Lewis Response to Reviewers.docx [file pone.0236839.s016.docx]

We thank the editors and reviewers for their comments. We have addressed them below and modified the manuscript accordingly. We believe that these suggestions improve the manuscript dramatically, and make the story more complete and readable. We have written our responses in red to make it easy to find our additions.

Journal Requirements:

When submitting your revision, we need you to address these additional requirements.

1. Please ensure that your manuscript meets PLOS ONE's style requirements, including those for file naming. The PLOS ONE style templates can be found at <https://journals.plos.org/plosone/s/file?id=wjVg/PLOSOne_formatting_sample_main_body.pdf> and <https://journals.plos.org/plosone/s/file?id=ba62/PLOSOne_formatting_sample_title_authors_affiliations.pdf>

We have amended these requirements.

2. Please note that PLOS does not permit references to “data not shown.” Authors should provide the relevant data within the manuscript, the Supporting Information files, or in a public repository. If the data are not a core part of the research study being presented, we ask that authors remove any references to these data.

As these statements were not integral to our story we removed them.

3. Please provide additional information about the K562 and HEK293T cells used in this work, including source, history, culture conditions and any quality control testing procedures (authentication, characterisation, and mycoplasma testing). For more information, please see <http://journals.plos.org/plosone/s/submission-guidelines#loc-cell-lines>.

We have added the following information to the manuscript:

K562 cells were obtained from the American Type Tissue Collection (ATCC, catalog number CCL-243) and maintained in RPMI 1640 media (Corning) with 10% fetal bovine serum (FBS), 1% penicillin/streptomycin, and 1% L-glutamine. Normal primary human peripheral blood mononuclear cells (PBMCs) were obtained from ATCC (catalog number PCS-800-011) and maintained in RPMI 1640 media (Corning) with 10% fetal bovine serum (FBS), 1% penicillin/streptomycin, and 1% L-glutamine. HEK293FT cells were obtained from ATCC (catalog number CRL-3216) and maintained in DMEM (Corning) with 10% FBS, 1% penicillin/streptomycin, and 1% L-glutamine. All mammalian cell lines were maintained at 37°C with 5% CO_2_. Primary Zebrafish Kidney Stromal (ZKS) cells were maintained and cultured as previously described[39]. All cell lines were monitored visually to confirm their morphology and tested for mycoplasma contamination via PCR analysis before use.

4. To comply with PLOS ONE submission guidelines, in your Methods section, please provide additional information regarding your statistical analyses. For more information on PLOS ONE's expectations for statistical reporting, please see <https://journals.plos.org/plosone/s/submission-guidelines.#loc-statistical-reporting>.”

We have added the following statement and are following your policies in the revised manuscript.

**Statistical methods**

Statistical analyses were performed in Microsoft Excel. To discern statistical difference, data were analyzed using an unpaired two-tailed Student’s T test assuming unequal variance. All data from these studies are supplied in Supporting Information.

5. At this time, we request that you  please report additional details in your Methods section regarding animal care, as per our editorial guidelines:

(1) Please state the source of the zebrafish used in the study

(2) Please provide details of animal welfare (e.g., shelter, food, water, environmental enrichment)

Thank you for your attention to these requests.

We added the following to the revised manuscript:

**Zebrafish husbandry and care**

Zebrafish were mated, staged, and raised as described[40] and maintained in accordance with California State University (CSU), Chico Institutional Animal Care and Use Committee (IACUC) guidelines. Personnel were trained in animal care by taking the online Citi Program training course entitled “Working With Zebrafish (*Danio rerio*) in Research Settings” ([https://www.citiprogram.org](https://www.citiprogram.org/index.cfm?pageID=154&icat=0&clear=1)). AB wild type (wt) fish were utilized for this study. Zebrafish were housed in a 700L recirculating zebrafish aquarium system (Aquatic Enterprises, Seattle, WA) regulated by a Profilux 3 Outdoor module that regulated salinity, pH, and temperature (GHL International, Kaiserslautern, Germany) 24-hours-a day. The facility was illuminated on a 14-hour light/ 10-hour dark cycle. Zebrafish were fed once a day with hatched brine shrimp (Brine Shrimp Direct, Ogden, UT) and once a day with Gemma micro 300 (Skretting, Westbrook, ME).

**Zebrafish toxicity**

Adult zebrafish (6-months-old), raised at our animal facility at CSU Chico, were individually placed in a 500 mL breeding tank with 15 μM of NHD2-15 (n=12, 6 males and 6 females), DMSO vehicle control (n=8, 4 males and 4 females), or 2 μM imatinib (n=8, 4 males and 4 females) and incubated for 3 days. Another experiment utilized 30 μM of NHD2-15 (n=8, 4 males and 4 females), and another experiment was performed whereby 30 μM of NHD2-15 (n=4, 2 males and 2 females) was added to the water for two hours before placing the fish into fresh system water. Every 24 h fish were fed. Every 2 hours fish were analyzed for signs of distress, including erratic swimming, ruffled gills and fins, lack of movement, and labored breathing. If fish showed any signs of distress they would have been immediately euthanized by an overdose of tricane methanesulfonate (MS-222), but that never occurred.

6. PLOS ONE now requires that authors provide the original uncropped and unadjusted images underlying all blot or gel results reported in a submission’s figures or Supporting Information files. This policy and the journal’s other requirements for blot/gel reporting and figure preparation are described in detail at <https://journals.plos.org/plosone/s/figures#loc-blot-and-gel-reporting-requirements> and <https://journals.plos.org/plosone/s/figures#loc-preparing-figures-from-image-files>. When you submit your revised manuscript, please ensure that your figures adhere fully to these guidelines and provide the original underlying images for all blot or gel data reported in your submission. See the following link for instructions on providing the original image data: <https://journals.plos.org/plosone/s/figures#loc-original-images-for-blots-and-gels>.

In your cover letter, please note whether your blot/gel image data are in Supporting Information or posted at a public data repository, provide the repository URL if relevant, and provide specific details as to which raw blot/gel images, if any, are not available. Email us at [plosone@plos.org](mailto:plosone@plos.org) if you have any questions.

Thank you for this note. We’ve now included the requested blots in the Supporting Information.

7. Thank you for stating the following in the Competing Interests section:

'D.L.S. is a scientific consultant and has received compensation from Finless Foods,
Inc. and Xytogen Biotechnology. All other authors declare no competing interests.'

Please confirm that this does not alter your adherence to all PLOS ONE policies on sharing data and materials, by including the following statement: "This does not alter our adherence to  PLOS ONE policies on sharing data and materials.” (as detailed online in our guide for authors [http://journals.plos.org/plosone/s/competing-interests](about:blank)).  If there are restrictions on sharing of data and/or materials, please state these. Please note that we cannot proceed with consideration of your article until this information has been declared.

We have amended the statement to read:

D.L.S. is a scientific consultant and has received compensation from Finless Foods, Inc. and Xytogen Biotech, Inc. This does not alter our adherence to PLOS ONE policies on sharing data and materials. All other authors declare no competing interests.

Please include your updated Competing Interests statement in your cover letter; we will change the online submission form on your behalf.

We have amended this in our cover letter as well.

8. Please include captions for your Supporting Information files at the end of your manuscript, and update any in-text citations to match accordingly. Please see our Supporting Information guidelines for more information: [http://journals.plos.org/plosone/s/supporting-information](about:blank).

We have amended this in the revised version.


Reviewers' comments:

Reviewer's Responses to Questions

**Comments to the Author**

1. Is the manuscript technically sound, and do the data support the conclusions?

The manuscript must describe a technically sound piece of scientific research with data that supports the conclusions. Experiments must have been conducted rigorously, with appropriate controls, replication, and sample sizes. The conclusions must be drawn appropriately based on the data presented. 

Reviewer #1: Partly

Hopefully the reviewer agrees that with the additional requested raw data and statistical information that the conclusions are sound.

2. Has the statistical analysis been performed appropriately and rigorously? 

Reviewer #1: No

We apologize, and did not realize that all these raw data should be supplied in the manuscript. We have added these analyses into the Supporting Information.

3. Have the authors made all data underlying the findings in their manuscript fully available?

The [PLOS Data policy](http://www.plosone.org/static/policies.action#sharing) requires authors to make all data underlying the findings described in their manuscript fully available without restriction, with rare exception (please refer to the Data Availability Statement in the manuscript PDF file). The data should be provided as part of the manuscript or its supporting information, or deposited to a public repository. For example, in addition to summary statistics, the data points behind means, medians and variance measures should be available. If there are restrictions on publicly sharing data—e.g. participant privacy or use of data from a third party—those must be specified.

Reviewer #1: No

Again, we apologize for this oversight. Raw data is now provided in the Supporting Information.

4. Is the manuscript presented in an intelligible fashion and written in standard English?

PLOS ONE does not copyedit accepted manuscripts, so the language in submitted articles must be clear, correct, and unambiguous. Any typographical or grammatical errors should be corrected at revision, so please note any specific errors here.

Reviewer #1: Yes

5. Review Comments to the Author

Please use the space provided to explain your answers to the questions above. You may also include additional comments for the author, including concerns about dual publication, research ethics, or publication ethics. (Please upload your review as an attachment if it exceeds 20,000 characters)

Reviewer #1: In this manuscript, Lewis and coworkers present a new small-molecule inhibitor of GRB2, a downstream effector of the important leukemia oncogene BCR-Abl1. The presented results are novel and interesting, the methodologies are scientifically sound. Overall I think the paper should be a good fit for PLOS ONE, but some of its conclusions/claims are weakly supported, therefore the authors should thoroughly revise it based on the following points.

Thanks to the reviewer for these helpful suggestions. We believe that the manuscript is much improved due to their constructive criticism.

1. The article seems to merge results from two independent studies: one for optimizing a novel synthetic reaction to yield a specific set of molecules, and one for testing some molecules against a specific oncotarget (GRB2). It is unclear how these studies were linked or what was the motivation to test the compounds against GRB2 in particular (or vice versa, to test these particular compounds against GRB2), even though the authors mention that NHD2-15 was "designed towards the SH2 domain of a specific protein". How was it designed? Did the authors establish a structural basis for its binding to the GRB2 SH2 domain (via ligand docking or similar computational methodologies)? Or were there similar compounds reported as SH2 inhibitors? Please elaborate on this in the manuscript.

This manuscript appears to be two different stories, but it really isn’t. This manuscript was a collaboration between Dr. Arpin (a medicinal chemist in the Chemistry department) and Dr. Stachura (a biologist that studies blood development and leukemia in the Biology department). We are at a primarily undergraduate institution and were exploring the idea of Dr. Arpin making these compounds in her chemistry classroom, and Dr. Stachura testing them on cancer cells in his immunology classroom. We could certainly break these stories up, presenting the medicinal chemistry part of the story in one manuscript and the studies on leukemia in a different manuscript. But, due to the fact that NHD2-15 performed much better than the other compounds, we chose to focus on that compound and fuse the two stories. Plus, we thought it would be of interest to a larger and more diverse readership, as it is a true synergy of chemistry and biology.

We do agree that we should at least explain the rationale of the drug’s design. We have added S3 Fig and descriptions to the manuscript. See below:

Design of the furo-quinoxaline scaffold of our novel antagonists was inspired by a previously studied SH2 domain-binder of a GRB protein[47] (S3 Fig). This compound has a central quinoxaline, and computational modeling revealed that the 2-hydroxyl and α-hydrogen off the 3-position of the quinoxaline adopt a planar conformation in the binding site. To increase the overall rigidity and ensure this planarity, we cyclized this central core of the compound, which led to our furo-quinoxaline scaffold.

2. Important details about statistical testing are left out. What statistical tests were applied to establish statistical significance of differences? Also, the authors claim that "the combination of NHD2-15 with imatinib significantly killed K562 cells better when compared to cells exposed to imatinib alone.", but on Figure 8, it is not reported whether these differences (1st striped column vs rest of striped columns) are significant or not.

We have added this information to the manuscript. And, we show the significance in the revised manuscript.

**Statistical Methods**

Statistical analyses were performed in Microsoft Excel. To discern statistical difference, data were analyzed using an unpaired two-tailed Student’s T test assuming unequal variance. All data from these studies are supplied in Supporting Information.

3. Non-toxicity was established by testing the smallest effective concentration (15uM) in zebrafish. However, the Kd of NHD2-15 to GRB2 is significantly higher (119uM) and the reported data do not suggest that 15uM would be a sufficient therapeutic concentration in vivo. Combined with the fact that the other three (structurally related) compounds were toxic to zebrafish, I think that non-toxicity should be established at a higher concentration.

We originally started with 15 uM, as even at that concentration K562 cells are significantly killed. Also, it takes a large amount of drug to do these studies. So, we started at the lowest concentration possible.

It is hard to determine the *in vivo* therapeutic concentration, especially using the zebrafish as a model system. Likely human patients will have the drug delivered differently for treatment, and we don’t know its *in vivo* half-life, organ specificity, etc. Sadly, there is no good CML model available for fish, so we couldn’t test it on a real tumor in an animal- that would be ideal. Our original idea was to use this toxicity assay to show that this drug was specific for GRB2, and wouldn’t kill healthy cells (as we indicate in the PBMC and ZKS studies). That being said, 30 uM is toxic to the fish after 2 hours of exposure. We present these data in S2B Fig. Basically, 30 uM is fine for 2 hours of exposure (S2C Fig.), so that might be the dose that is used therapeutically, just not with constant exposure. We have added discussion of these issues to the **Results** and **Discussion** section of the manuscript.

4. As per journal guidelines on data availability, the raw data should be published in a tabular format as supplementary data.

We apologize, and did not know that. All data has been added to the Supporting Information section of the manuscript.

Minor:

- Figure 2 x axis title is confusing, as the native peptide is not an antagonist of GRB2, please revise.

We agree and have changed it to simply read “concentration (uM)”.

- Figure 6 takes up space unnecessarily: it would be enough to state that none of the zebrafish were killed under the specific conditions.

We agree and have put these data into S2 Fig.

- Results/Library synthesis section: "Finally, deprotonation of 2 with sodium hydride..." I believe it is the beta-diketone that is deprotonated, please double-check this sentence.

We apologize for that oversight. The reviewer is correct and it has been corrected in the revised manuscript.

6. PLOS authors have the option to publish the peer review history of their article ([what does this mean?](https://journals.plos.org/plosone/s/editorial-and-peer-review-process#loc-peer-review-history)). If published, this will include your full peer review and any attached files.


If you choose “no”, your identity will remain anonymous but your review may still be made public.


**Do you want your identity to be public for this peer review?** For information about this choice, including consent withdrawal, please see our [Privacy Policy](https://www.plos.org/privacy-policy).

Reviewer #1: No

We have no concerns about the review and responses being made public, if the reviewer and editor so choose.

[NOTE: If reviewer comments were submitted as an attachment file, they will be attached to this email and accessible via the submission site. Please log into your account, locate the manuscript record, and check for the action link "View Attachments". If this link does not appear, there are no attachment files.]

While revising your submission, please upload your figure files to the Preflight Analysis and Conversion Engine (PACE) digital diagnostic tool, <https://pacev2.apexcovantage.com/>. PACE helps ensure that figures meet PLOS requirements. To use PACE, you must first register as a user. Registration is free. Then, login and navigate to the UPLOAD tab, where you will find detailed instructions on how to use the tool. If you encounter any issues or have any questions when using PACE, please email PLOS at [figures@plos.org](mailto:figures@plos.org). Please note that Supporting Information files do not need this step.
