# Supplementary material for: NHD2-15, a novel antagonist of Growth Factor Receptor-Bound Protein-2 (GRB2), inhibits leukemic proliferation
Source: PLoS One. 2020 Aug 11;15(8):e0236839. doi: 10.1371/journal.pone.0236839 (PMC7418987; doi:10.1371/journal.pone.0236839)
Supplement: Peer review history

# Peer review: Decision Letter 0

**DOI:** 10.1371/journal.pone.0236839.r001

**Authors:** Laszlo Buday

16 Jun 2020

PONE-D-20-13006

NHD2-15, a Novel Antagonist of Growth Factor Receptor-Bound Protein-2 (GRB2), Inhibits Leukemic Proliferation

PLOS ONE

Dear Dr. Stachura,

Thank you for submitting your manuscript to PLOS ONE. After careful consideration, we feel that it has merit but does not fully meet PLOS ONE’s publication criteria as it currently stands. Therefore, we invite you to submit a revised version of the manuscript that addresses the points raised during the review process.

Your manuscript was reviewed by a knowledgeable referee in the area. As noted in the attached comments, the reviewer found merit in your findings, however, he/she identified a number of important issues that must be addressed before the work can be considered further for potential publication. Please address these issues in a thoughtful and complete manner and submit the revised manuscript for further consideration.  

Please submit your revised manuscript by Jul 31 2020 11:59PM. If you will need more time than this to complete your revisions, please reply to this message or contact the journal office at plosone@plos.org. When you're ready to submit your revision, log on to https://www.editorialmanager.com/pone/ and select the 'Submissions Needing Revision' folder to locate your manuscript file.

Please include the following items when submitting your revised manuscript:

- A rebuttal letter that responds to each point raised by the academic editor and reviewer(s). You should upload this letter as a separate file labeled 'Response to Reviewers'.
- A marked-up copy of your manuscript that highlights changes made to the original version. You should upload this as a separate file labeled 'Revised Manuscript with Track Changes'.
- An unmarked version of your revised paper without tracked changes. You should upload this as a separate file labeled 'Manuscript'.

If you would like to make changes to your financial disclosure, please include your updated statement in your cover letter. Guidelines for resubmitting your figure files are available below the reviewer comments at the end of this letter.

If applicable, we recommend that you deposit your laboratory protocols in protocols.io to enhance the reproducibility of your results. Protocols.io assigns your protocol its own identifier (DOI) so that it can be cited independently in the future. For instructions see: http://journals.plos.org/plosone/s/submission-guidelines#loc-laboratory-protocols

We look forward to receiving your revised manuscript.

Kind regards,

Laszlo Buday

Academic Editor

PLOS ONE

Journal Requirements:

When submitting your revision, we need you to address these additional requirements.

1. Please ensure that your manuscript meets PLOS ONE's style requirements, including those for file naming. The PLOS ONE style templates can be found at https://journals.plos.org/plosone/s/file?id=wjVg/PLOSOne_formatting_sample_main_body.pdf and https://journals.plos.org/plosone/s/file?id=ba62/PLOSOne_formatting_sample_title_authors_affiliations.pdf

2. Please note that PLOS does not permit references to “data not shown.” Authors should provide the relevant data within the manuscript, the Supporting Information files, or in a public repository. If the data are not a core part of the research study being presented, we ask that authors remove any references to these data.

3. Please provide additional information about the K562 and HEK293T cells used in this work, including source, history, culture conditions and any quality control testing procedures (authentication, characterisation, and mycoplasma testing). For more information, please see http://journals.plos.org/plosone/s/submission-guidelines#loc-cell-lines.

4. To comply with PLOS ONE submission guidelines, in your Methods section, please provide additional information regarding your statistical analyses. For more information on PLOS ONE's expectations for statistical reporting, please see https://journals.plos.org/plosone/s/submission-guidelines.#loc-statistical-reporting.”

5. At this time, we request that you  please report additional details in your Methods section regarding animal care, as per our editorial guidelines:

(1) Please state the source of the zebrafish used in the study  

(2) Please provide details of animal welfare (e.g., shelter, food, water, environmental enrichment)

Thank you for your attention to these requests.

6. PLOS ONE now requires that authors provide the original uncropped and unadjusted images underlying all blot or gel results reported in a submission’s figures or Supporting Information files. This policy and the journal’s other requirements for blot/gel reporting and figure preparation are described in detail at https://journals.plos.org/plosone/s/figures#loc-blot-and-gel-reporting-requirements and https://journals.plos.org/plosone/s/figures#loc-preparing-figures-from-image-files. When you submit your revised manuscript, please ensure that your figures adhere fully to these guidelines and provide the original underlying images for all blot or gel data reported in your submission. See the following link for instructions on providing the original image data: https://journals.plos.org/plosone/s/figures#loc-original-images-for-blots-and-gels.

In your cover letter, please note whether your blot/gel image data are in Supporting Information or posted at a public data repository, provide the repository URL if relevant, and provide specific details as to which raw blot/gel images, if any, are not available. Email us at plosone@plos.org if you have any questions.

7. Thank you for stating the following in the Competing Interests section:

'D.L.S. is a scientific consultant and has received compensation from Finless Foods,

Inc. and Xytogen Biotechnology. All other authors declare no competing interests.'

Please confirm that this does not alter your adherence to all PLOS ONE policies on sharing data and materials, by including the following statement: "This does not alter our adherence to  PLOS ONE policies on sharing data and materials.” (as detailed online in our guide for authors http://journals.plos.org/plosone/s/competing-interests).  If there are restrictions on sharing of data and/or materials, please state these. Please note that we cannot proceed with consideration of your article until this information has been declared.

Please include your updated Competing Interests statement in your cover letter; we will change the online submission form on your behalf.

Please know it is PLOS ONE policy for corresponding authors to declare, on behalf of all authors, all potential competing interests for the purposes of transparency. PLOS defines a competing interest as anything that interferes with, or could reasonably be perceived as interfering with, the full and objective presentation, peer review, editorial decision-making, or publication of research or non-research articles submitted to one of the journals. Competing interests can be financial or non-financial, professional, or personal. Competing interests can arise in relationship to an organization or another person. Please follow this link to our website for more details on competing interests: http://journals.plos.org/plosone/s/competing-interests

8. Please include captions for your Supporting Information files at the end of your manuscript, and update any in-text citations to match accordingly. Please see our Supporting Information guidelines for more information: http://journals.plos.org/plosone/s/supporting-information.

Additional Editor Comments (if provided):

[Note: HTML markup is below. Please do not edit.]

Reviewers' comments:

Reviewer's Responses to Questions

**Comments to the Author**

1. Is the manuscript technically sound, and do the data support the conclusions?

The manuscript must describe a technically sound piece of scientific research with data that supports the conclusions. Experiments must have been conducted rigorously, with appropriate controls, replication, and sample sizes. The conclusions must be drawn appropriately based on the data presented. 

Reviewer #1: Partly

**********

2. Has the statistical analysis been performed appropriately and rigorously? 

Reviewer #1: No

**********

3. Have the authors made all data underlying the findings in their manuscript fully available?

The PLOS Data policy requires authors to make all data underlying the findings described in their manuscript fully available without restriction, with rare exception (please refer to the Data Availability Statement in the manuscript PDF file). The data should be provided as part of the manuscript or its supporting information, or deposited to a public repository. For example, in addition to summary statistics, the data points behind means, medians and variance measures should be available. If there are restrictions on publicly sharing data—e.g. participant privacy or use of data from a third party—those must be specified.

Reviewer #1: No

**********

4. Is the manuscript presented in an intelligible fashion and written in standard English?

PLOS ONE does not copyedit accepted manuscripts, so the language in submitted articles must be clear, correct, and unambiguous. Any typographical or grammatical errors should be corrected at revision, so please note any specific errors here.

Reviewer #1: Yes

**********

5. Review Comments to the Author

Please use the space provided to explain your answers to the questions above. You may also include additional comments for the author, including concerns about dual publication, research ethics, or publication ethics. (Please upload your review as an attachment if it exceeds 20,000 characters)

Reviewer #1: In this manuscript, Lewis and coworkers present a new small-molecule inhibitor of GRB2, a downstream effector of the important leukemia oncogene BCR-Abl1. The presented results are novel and interesting, the methodologies are scientifically sound. Overall I think the paper should be a good fit for PLOS ONE, but some of its conclusions/claims are weakly supported, therefore the authors should thoroughly revise it based on the following points.

1. The article seems to merge results from two independent studies: one for optimizing a novel synthetic reaction to yield a specific set of molecules, and one for testing some molecules against a specific oncotarget (GRB2). It is unclear how these studies were linked or what was the motivation to test the compounds against GRB2 in particular (or vice versa, to test these particular compounds against GRB2), even though the authors mention that NHD2-15 was "designed towards the SH2 domain of a specific protein". How was it designed? Did the authors establish a structural basis for its binding to the GRB2 SH2 domain (via ligand docking or similar computational methodologies)? Or were there similar compounds reported as SH2 inhibitors? Please elaborate on this in the manuscript.

2. Important details about statistical testing are left out. What statistical tests were applied to establish statistical significance of differences? Also, the authors claim that "the combination of NHD2-15 with imatinib significantly killed K562 cells better when compared to cells exposed to imatinib alone.", but on Figure 8, it is not reported whether these differences (1st striped column vs rest of striped columns) are significant or not.

3. Non-toxicity was established by testing the smallest effective concentration (15uM) in zebrafish. However, the Kd of NHD2-15 to GRB2 is significantly higher (119uM) and the reported data do not suggest that 15uM would be a sufficient therapeutic concentration in vivo. Combined with the fact that the other three (structurally related) compounds were toxic to zebrafish, I think that non-toxicity should be established at a higher concentration.

4. As per journal guidelines on data availability, the raw data should be published in a tabular format as supplementary data.

Minor:

- Figure 2 x axis title is confusing, as the native peptide is not an antagonist of GRB2, please revise.

- Figure 6 takes up space unnecessarily: it would be enough to state that none of the zebrafish were killed under the specific conditions.

- Results/Library synthesis section: "Finally, deprotonation of 2 with sodium hydride..." I believe it is the beta-diketone that is deprotonated, please double-check this sentence.

**********

6. PLOS authors have the option to publish the peer review history of their article (what does this mean?). If published, this will include your full peer review and any attached files.

If you choose “no”, your identity will remain anonymous but your review may still be made public.

**Do you want your identity to be public for this peer review?** For information about this choice, including consent withdrawal, please see our Privacy Policy.

Reviewer #1: No

[NOTE: If reviewer comments were submitted as an attachment file, they will be attached to this email and accessible via the submission site. Please log into your account, locate the manuscript record, and check for the action link "View Attachments". If this link does not appear, there are no attachment files.]

While revising your submission, please upload your figure files to the Preflight Analysis and Conversion Engine (PACE) digital diagnostic tool, https://pacev2.apexcovantage.com/. PACE helps ensure that figures meet PLOS requirements. To use PACE, you must first register as a user. Registration is free. Then, login and navigate to the UPLOAD tab, where you will find detailed instructions on how to use the tool. If you encounter any issues or have any questions when using PACE, please email PLOS at figures@plos.org. Please note that Supporting Information files do not need this step.

# Author comment: Author response to Decision Letter 0

**DOI:** 10.1371/journal.pone.0236839.r002

10 Jul 2020

Please see enclosed "Response to Reviewers" File

Attachment Submitted filename: Lewis Response to Reviewers.docx Click here for additional data file.

# Editor report: Decision Letter 1

**DOI:** 10.1371/journal.pone.0236839.r003

**Authors:** Laszlo Buday

15 Jul 2020

NHD2-15, a Novel Antagonist of Growth Factor Receptor-Bound Protein-2 (GRB2), Inhibits Leukemic Proliferation

PONE-D-20-13006R1

Dear Dr. Stachura,

We’re pleased to inform you that your manuscript has been judged scientifically suitable for publication and will be formally accepted for publication once it meets all outstanding technical requirements.

Within one week, you’ll receive an e-mail detailing the required amendments. When these have been addressed, you’ll receive a formal acceptance letter and your manuscript will be scheduled for publication.

An invoice for payment will follow shortly after the formal acceptance. To ensure an efficient process, please log into Editorial Manager at http://www.editorialmanager.com/pone/, click the 'Update My Information' link at the top of the page, and double check that your user information is up-to-date. If you have any billing related questions, please contact our Author Billing department directly at authorbilling@plos.org.

If your institution or institutions have a press office, please notify them about your upcoming paper to help maximize its impact. If they’ll be preparing press materials, please inform our press team as soon as possible -- no later than 48 hours after receiving the formal acceptance. Your manuscript will remain under strict press embargo until 2 pm Eastern Time on the date of publication. For more information, please contact onepress@plos.org.

Kind regards,

Laszlo Buday

Academic Editor

PLOS ONE

Additional Editor Comments (optional):

Reviewers' comments:

# Editor report: Acceptance letter

**DOI:** 10.1371/journal.pone.0236839.r004

**Authors:** Laszlo Buday

16 Jul 2020

PONE-D-20-13006R1 

NHD2-15, a novel antagonist of Growth Factor Receptor-Bound Protein-2 (GRB2), inhibits leukemic proliferation 

Dear Dr. Stachura:

I'm pleased to inform you that your manuscript has been deemed suitable for publication in PLOS ONE. Congratulations! Your manuscript is now with our production department. 

If your institution or institutions have a press office, please let them know about your upcoming paper now to help maximize its impact. If they'll be preparing press materials, please inform our press team within the next 48 hours. Your manuscript will remain under strict press embargo until 2 pm Eastern Time on the date of publication. For more information please contact onepress@plos.org.

If we can help with anything else, please email us at plosone@plos.org. 

Thank you for submitting your work to PLOS ONE and supporting open access. 

Kind regards, 

PLOS ONE Editorial Office Staff

on behalf of

Professor Laszlo Buday 

Academic Editor

PLOS ONE
